# ACE2 negatively regulates the Warburg effect and suppresses hepatocellular carcinoma progression via reducing ROS-HIF1α activity

**DOI:** 10.7150/ijbs.81498

**Published:** 2023-05-11

**Authors:** Fangyuan Dong, Hui Li, Limin Liu, Lin-Li Yao, Jiaofeng Wang, Danni Xiang, Jianxia Ma, Gansheng Zhang, Shan Zhang, Jun Li, Shu-Heng Jiang, Xiaona Hu, Jie Chen, Zhijun Bao

**Affiliations:** 1 Department of Gastroenterology, Huadong Hospital, Shanghai Medical College, Fudan University, Shanghai 200040, P.R. China.; 2 State Key Laboratory of Oncogenes and Related Genes, Shanghai Cancer Institute, Ren Ji Hospital, School of Medicine, Shanghai Jiao Tong University, Shanghai 200240, P.R. China.; 3 Shanghai Key Laboratory of Clinical Geriatric Medicine, Shanghai 200040, P.R. China.; 4 National Clinical Research Center for Aging and Medicine, Shanghai 200040, P.R. China.; 5 Department of Geriatrics, Huadong Hospital, Shanghai Medical College, Fudan University Shanghai 200040, P.R. China.; 6 Department of Oral pathology, Ninth People's Hospital, School of Medicine, Shanghai Jiao Tong University, Shanghai 200011, P.R. China.

**Keywords:** Warburg effect, Liver cancer, Hypoxia-inducible factor, Metabolic reprogramming

## Abstract

Aerobic glycolysis has pleiotropic roles in the pathogenesis of hepatocellular carcinoma (HCC). Emerging studies revealed key promoters of aerobic glycolysis, however, little is known about its negative regulators in HCC. In this study, an integrative analysis identifies a repertoire of differentially expressed genes (*DNASE1L3*, *SLC22A1*, *ACE2*, *CES3*, *CCL14*, *GYS2*, *ADH4*, and *CFHR3*) that are inversely associated with the glycolytic phenotype in HCC. ACE2, a member of the rennin-angiotensin system, is revealed to be downregulated in HCC and predicts a poor prognosis. ACE2 overexpression significantly inhibits the glycolytic flux as evidenced by reduced glucose uptake, lactate release, extracellular acidification rate, and the expression of glycolytic genes. Opposite results are noticed in loss-of-function studies. Mechanistically, ACE2 metabolizes Ang II to Ang-(1-7), which activates Mas receptor and leads to the phosphorylation of Src homology 2-containing inositol phosphatase 2 (SHP-2). SHP2 activation further blocks reactive oxygen species (ROS)-HIF1α signaling. Addition of Ang-(1-7) or the antioxidant N-acetylcysteine compromises *in vivo* additive tumor growth and aerobic glycolysis induced by ACE2 knockdown. Moreover, growth advantages afforded by ACE2 knockdown are largely glycolysis-dependent. In clinical settings, a close link between ACE2 expression and HIF1α or the phosphorated level of SHP2 is found. Overexpression of ACE2 significantly retards tumor growth in patient-derived xenograft model. Collectively, our findings suggest that ACE2 is a negative glycolytic regulator, and targeting the ACE2/Ang-(1-7)/Mas receptor/ROS/HIF1α axis may be a promising therapeutic strategy for HCC treatment.

## Introduction

In normal cells, glycolysis converts glucose to pyruvate, which enters the tricarboxylic acid cycle (TCA) to produce adenosine triphosphate (ATP). Under pathological conditions such as cancer, fast-growing cancer cells preferentially convert glucose to lactate even in the presence of sufficient oxygen, a phenomenon termed the Warburg effect or aerobic glycolysis 1-3. Reprogrammed metabolic reprogramming is widely observed in human cancers such as hepatocellular carcinoma (HCC) and is emerged as a hallmark of cancer 4, 5. Cancer cells tend to utilize glycolysis rather than oxidative phosphorylation to adapt to the hypoxic tumor microenvironment (TME) and this process largely depends on the abnormally expressed glycolytic genes, such as glucose transporter 1 (GLUT1), hexokinase 2 (HK2), liver-type phosphofructokinase (PFKL), and lactate dehydrogenase A (LDHA) 6-8. Accumulated studies have revealed a close link between aerobic glycolysis and tumor progression, including but not limited to tumor growth, metastasis, apoptosis, autophagy, stemness, and drug resistance 9. Notably, tumors with a higher glycolytic capacity are associated with a poorer clinical outcome in HCC patients 10. Therefore, targeting aerobic glycolysis is a promising strategy and of great importance for cancer treatment.

Previously, many oncogenes have been demonstrated to be key players in the process of aerobic glycolysis, especially hypoxia-inducible factor 1 alpha (HIF1α), AKT, c-Myc, FOXK1/2, and SIX1 10-14. In HCC, many aberrantly expressed genes are documented as positive glycolysis regulators 15-17. For instance, PARP14 enhances aerobic glycolysis via inhibition of JNK1-dependent pyruvate kinase M2 (PKM2) phosphorylation and activation 16 and the fatty acid receptor CD36 exerts a stimulatory effect on HCC growth and metastasis in a glycolysis-dependent manner 15. Recently, we demonstrated that hypoxia-induced MAP17 increases the glycolytic flux of HCC cells via the regulation of reactive oxygen species (ROS) signaling 18. Although much progress has uncovered the oncogenic drivers of aerobic glycolysis in HCC, the negative regulators of aerobic glycolysis and corresponding molecular mechanisms are incompletely understood. From the therapeutic point of view, pharmacological activation of the negative regulators (tumor suppressors) of glucose metabolism may be an alternate strategy for cancer treatment.

Recently, angiotensin-converting enzyme 2 (ACE2) is known as a host cell receptor for severe acute respiratory syndrome coronavirus 2 (SARS-CoV-2) 19. As an arm of the renin-angiotensin (Ang) system (RAS), ACE2 can convert angiotensin II (Ang II) to Ang-(1-7), a heptapeptide acting through the Mas receptor 20-22. The ACE2/Ang(1-7)/Mas receptor axis is frequently downregulated in human cancers and plays tumor-suppressive roles 23-25. For example, ACE2 inhibits angiogenesis via downregulating the VEGFa/VEGFR2/ERK pathway in breast cancer 26. Activation of the ACE2/Ang-(1-7) axis in clear cell renal cell carcinoma (ccRCC) abrogates tumor resistance to VEGFR inhibitors 27. However, little is known about the link between ACE2 and glycolytic metabolism in cancers.

Given that the positive regulators of aerobic glycolysis are well documented in HCC, we aimed to identify the negative regulators of HCC glycolytic metabolism. In the present study, ACE2 was revealed to be a candidate that is inversely associated with HCC glycolysis. *In vitro* and *in vivo* experiments showed that ACE2 exerts an antitumor effect on HCC via Ang(1-7)/Mas receptor axis. Furthermore, phosphorylation of Src homology 2-containing inositol phosphatase 2 (SHP-2), ROS generation, and HIF1α signaling were demonstrated to be the functional mediators of ACE2 in HCC.

## Materials and methods

### Cell lines, cell culture, and reagents

HCC cell lines (Huh-7, HCC-LM3, Hep3B, SMMC-7721, SNU-475, MHCC-97L, SK-Hep1, and SUN-389) and the nonmalignant liver cell lines (LO2 and THLE-2) were obtained from the Cell Bank of the Chinese Academy of Sciences (Shanghai, China) or American Type Culture Collection (ATCC; Manassas, VA, USA). Short tandem repeat profiling and mycoplasma contamination examination were done before cell experiments for each cell line. Cells were cultured with Dulbecco's modified Eagle's medium (Gibco, 11965092) or Roswell Park Memorial Institute 1640 medium (Gibco, 11875093), supplemented with 10% fetal bovine serum (FBS, Gibco) and 1% (v/v) streptomycin-penicillin (Sigma-Aldrich, Shanghai, China). All the cells used in this study were cultured in a humidified incubator at 37 °C with 5% CO_2_. The antioxidant N-acetylcysteine (NAC) (S1623), Angiotensin (1-7) (S9820), MLN-4760 (S8940), and Mas receptor inhibitor A-779 (E0039) were all purchased from Selleck (Shanghai, China).

### ACE2 gene expression analysis

The online database TIMER (https://cistrome.shinyapps.io/timer/) was employed to investigate the expression profiles of ACE2 across human cancers.

### Prognostic analysis

The online database Kaplan-Meier Plotter (https://kmplot.com/analysis/) was used to investigate the prognostic value of ACE2 in HCC. Data were derived from the TCGA cohort and sample grouping was made based on the median mRNA level of ACE2. Kaplan-Meier method was used to determine the prognostic value and the difference was analyzed by the log-rank test.

### Definition of glycolysis gene signature and sample grouping

A 16-gene expression signature including genes specific to glycolysis (*ALDOA*, *ALDOB*, *ENO1*, *ENO2*, *GAPDH*, *GPI*, *HK2*, *LDHA*, *PFKFB1*, *PFKP*, *PGAM1*, *PGAM2*, *PGK1*, *PKM2*, *SLC2A1*, and *TPI1*) was used for the generation of GLYCOLYSIS gene set. Gene set variation analysis (GSVA) was used to calculate the enrichment scores of GLYCOLYSIS gene set based on the expression data set of LIHC from TCGA. The patients were divided into GLYCOLYSIS-high and -low group by the median cutoff of GSVA scoring of GLYCOLYSIS gene set variation analysis. The differentially expressed genes (DEGs) between GLYCOLYSIS-high and -low group were identified using two-tailed Wilcoxon signed rank test and false discovery rate (FDR) correction procedure, and the fold change (log_2_FC) between the two groups was calculated.

### Gene set enrichment analysis (GSEA)

The publicly available TCGA-LIHC data was used to characterize the molecular differences in patients with high versus low ACE2 expression. Sample grouping was based on the median value of ACE2 expression, and the sample numbers were sufficient to produce statistically significant differences. GSEA was performed with the Hallmark gene sets. The signaling pathway with a false discovery rate (FDR) less than 0.25 and a P value less than 0.05 were considered significantly enriched.

### Cell transfection

For ACE2 knockdown experiments, two specific short hairpin RNAs (shRNAs) against ACE2 gene were synthesized by GenePharma (Shanghai, China). shRNA plasmids along with a three-plasmid system (pPACKH1-REV, pPACKH1-GAG, and pVSV-G) were transfected into HEK293T cells to generate lentivirus using Lipofectamine 2000 (Invitrogen, Carlsbad, CA, USA) according to the manufacturer's instructions. Lentivirus were collected and subjected to cell infection with polybrene (Sigma-Aldrich, H9268, St. Louis, MO). Stable shRNA-expressing cells were selected with 2 μg/ml puromycin for 2 weeks. For ACE2 overexpression experiments, the expression construct for human wild type ACE2 (ACE2^WT^) and enzymatic-dead ACE2 (ACE2^H505L^) was synthesized by GenePharma (Shanghai, China) and subcloned into the pGCMV/MCS/IRES/EGFP/Neo plasmid. Stable ACE2-expressing cells were generated by lentivirus infection and the overexpression efficiency was verified by Western blotting.

### Western blotting analysis

Whole-cell proteins were extracted using RIPA lysis buffer (P0013B, Beyotime, Shanghai, China) mixed with protease and phosphatase inhibitor cocktails (ab201119, Abcam, Shanghai, China). The protein concentration was measured by the BCA Protein Assay Kit (Pierce Biotechnology, USA) according to the manufacturer's instructions. The proteins were separated by sodium dodecyl sulfate-polyacrylamide gel electrophoresis and transferred to polyvinylidene difluoride membranes (PVDF; Millipore). The membranes were blocked with 5% bovine serum albumin (BSA) for one hour at room temperature and incubated with primary antibodies at 4 °C overnight. The following antibodies were used in this study: ACE2 (1:1000, Abcam, ab108252), HIF1α (1: 1000, Abcam, ab113642), p-P38 (1:1000, Cell Signaling Technology, #4511), P38 (1:1000, Cell Signaling Technology, #8690), p-p44/42 MAPK (1:1000, Cell Signaling Technology, #4370), p44/42 MAPK (1:2,000, Cell Signaling Technology, #4695), p-JNK (1:1000, Cell Signaling Technology, #9255), JNK (1:2,000, Cell Signaling Technology, #9252), p-P65 (1:1,000, Cell Signaling Technology, #3033), P65 (1:1000, Cell Signaling Technology, #8242), p-Akt (1:2,000, Cell Signaling Technology, #4060), Akt (1:1000, Cell Signaling Technology, #4685), p-SHP2 (1:1,000, Cell Signaling Technology, #5431), SHP2 (1:1000, Cell Signaling Technology, #3397), and β-actin (1:2000, Abcam, ab8226). On the second day, the membranes were incubated with HRP-conjugated secondary antibodies for 45 min at room temperature and visualized using an ECL chemiluminescence assay.

### Quantitative real-time PCR (qRT-PCR)

Total RNA was extracted from indicated HCC cell lines or xenograft tumor tissues using the RNAiso Plus reagent (Takara, Japan) and subjected to reverse transcription reaction using the PrimeScript RT-PCR kit (Takara, Japan). qRT-PCR was performed with SYBR Green (Takara, Japan) on a 7500 Real-time PCR system (Applied Biosystems, Inc. USA). The housekeeping gene ACTB was used as an internal control. The sequences of primers were shown as follows: ACE2 forward, 5′-CAAGAGCAAACGGTTGAACAC-3′; ACE2 reverse 5′-CCAGAGCCTCTCATTGTAGTCT-3′; SLC2A1 forward, 5′-ATTGGCTCCGGTATCGTCAAC-3′; SLC2A1 reverse, 5′-GCTCAGATAGGACATCCAGGGTA-3′; HK2 forward, 5′-TTGACCAGGAGATTGACATGGG-3′; HK2 reverse, 5′-CAACCGCATCAGGACCTCA-3′; ENO1 forward, 5′-TGGTGTCTATCGAAGATCCCTT-3′; ENO1 reverse, 5′-CCTTGGCGATCCTCTTTGG-3′; PFKL forward, 5′-GCTGGGCGGCACTATCATT-3′; PFKL reverse, 5′-TCAGGTGCGAGTAGGTCCG-3′; LDHA forward, 5′-ATGGCAACTCTAAAGGATCAGC-3′; LDHA reverse, 5′-CCAACCCCAACAACTGTAATCT-3′; PDK1 forward, 5′-GGATTGCCCATATCACGTCTTT-3′; PDK1 reverse, 5′-TCCCGTAACCCTCTAGGGAATA-3′; ACTB forward, 5′-ACTCGTCATACTCCTGCT-3′, ACTB reverse, 5′-GAAACTACCTTCAACTCC-3′.

### Measurement of glucose and lactate level

Glucose and lactate levels in the cell supernatant were detected as reported previously 29. The Amplex Red Glucose/Glucose Oxidase Assay Kit (A22189, Thermo Fisher Scientific, USA) and the Lactate Assay Kit (K607-100, BioVision, USA) were used to detect glucose and lactate levels, respectively. The acquired data were further normalized to the corresponding protein concentration of cell extracts. All the experiments were run in triplicate and repeated at least two times.

### Extracellular acidification rate (ECAR)

The Seahorse Bioscience XF96 Extracellular Flux Analyzer (Seahorse Bioscience, USA) was used to analyze ECAR in HCC cells upon different treatments. ECAR measurement was analyzed with Seahorse XF Cell Glycolysis Stress Test Kit (Seahorse Bioscience, USA) according to the manufacturer's protocols. In this study, 10 mM glucose, 0.5-1 μM oligomycin (Oligo), and 80 mM 2-deoxyglucose (2-DG) were used for ECAR detection. The acquired data were further normalized to the corresponding protein concentration of cell extracts.

### HIF1α transcriptional activity

The commercial HIF1α Transcription Factor Assay Kit (ab133104, Abcam) was used to determine the effect of ACE2/Mas receptor/NAC on HIF1α activity. In brief, nuclear extract lysates were harvested from indicated HCC cells by using a Nuclear Extraction Kit (#2900, Millipore), followed by HIF1α activity detection with the assay kit according to the manufacturer's protocols.

### Detection of reactive oxygen species (ROS)

For evaluating the level of cellular ROS, 1 × 10^4^ indicated HCC cells were seeded at a well of black 96-well plates and subjected to DCF-DA (10 mmol/L) staining in phenol red-free medium for 30 min at room temperature. Then the fluorescence intensity was detected immediately using a BioTek FLx800 Microplate Fluorescence Readers.

### Cell proliferation assay

HCC cells were seeded at 500 cells per well in 6-well plates. The culture medium was replaced every 2-3 days and cells were cultured for 10-14 days. At the end time point, HCC cells were fixed with 4% paraformaldehyde for 15 min and stained with 0.1% crystal violet for 20 min. After washing with PBS three times, the colonies were counted. All the experiments were run in triplicate and repeated at least two times.

### Cell apoptosis assay

The commercial Apo-ONE Homogeneous Caspase-3/7 Assay Kit (Promega, G7790, USA) was used to determine cell apoptosis. CellTiter-Blue (Promega, G8081) was used to evaluate cell numbers. At the endpoint of experiments, cell number and caspase-3/7 activity were monitored in the same sample. The Caspase-3/7 activity was calculated as the ratio of Apo-ONE/CellTiter-Blue signals according to the manufacturer's instructions. All the experiments were run in triplicate and repeated at least two times.

### Animal experiments

Male BALB/c nude mice aged six to eight weeks were purchased from Shanghai Jiesijie Laboratory Animal Technology Company. Mice were housed in pathogen-free conditions and maintained in a 12/12 h light-dark cycle with free access to standard food and tap water. To generate a subcutaneous xenograft model, 2 × 10^6^ indicated HCC cells were resuspended in 100 μL PBS and then implanted into the flanks of mice. The volume of xenograft tumors was monitored every 3 or 4 days. For patient-derived xenograft (PDX) model, two HCC samples were acquired and subjected for evaluation of ACE2 expression. For treatment of AAV (GenePharma, Shanghai, China), tumor-bearing mice were intratumorally injected with control AAV or AAV-oeACE2. Tumor volume was estimated as follows: tumor volume = length×width^2^/2. At the endpoint of the animal experiment, the mice were sacrificed and the xenograft tumors were isolated and weighed. This study was approved by the Research Ethics Committee of Huadong Hospital, Shanghai Medical College, Fudan University and carried out following the guidelines of the national animal protection and ethics institute.

### Immunohistochemistry

A microarray containing 202 matched HCC and nontumor tissues was used as reported previously 28. Immunohistochemical (IHC) analysis was carried out as reported previously 29. The following primary antibodies were used for IHC analysis: ACE2 (1:200, Abcam, ab108252), Ki67 (1:400, Cell Signaling Technology, #9449), cleaved caspase 3 (1:400, Cell Signaling Technology, #9661), HIF1α (1:200, Abcam, ab51608), and p-SHP2 (1:200, Abcam, ab75818). Scoring was conducted based on the percentage of positive staining cells and staining intensity as reported previously 30. “-” indicates no staining, “+” indicates weak staining, “++” indicates moderate staining, and “+++” indicates strong staining. “-” and “+” were defined as low ACE2 expression, while “++” and “+++” were defined as high ACE2 expression.

### Statistical analysis

All the data were presented as means ± SEM from at least three independent experiments. When comparing two independent groups, a two-tailed Student's t-test was used. When comparing two independent groups. Statistical analyses for more than two groups (parametric variables) were performed with a two-way analysis of variance (ANOVA) followed by post hoc Duncan tests. GraphPad Prism (GraphPad Software Inc., San Diego, CA) was used for statistical analyses. P-value less than 0.05 was considered statistically significant.

## Results

### Identification of negative regulators of aerobic glycolysis in HCC

**Figure 1:**
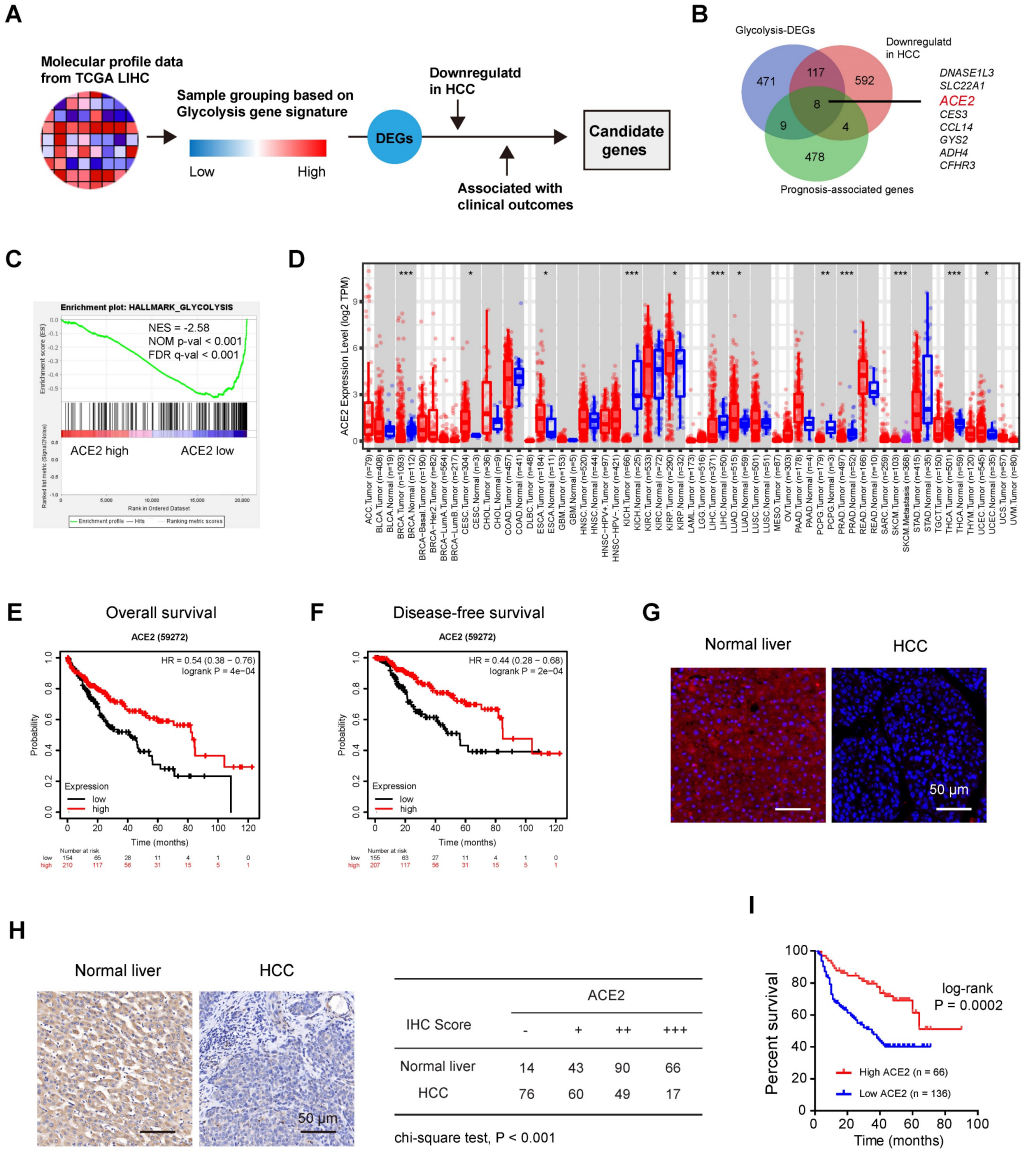
**Identification of negative regulators of aerobic glycolysis in HCC.** (**A**) Workflow for identifying differentially expressed genes (DEGs) related to glycolysis in HCC. (**B**) Genes that negatively linked to glycolysis, genes that downregulated in HCC, and prognosis-associated genes were merged. All the differentially expressed genes (DEGs) were identified with The Cancer Genome Atlas (TCGA) cohort. (**C**) Gene set enrichment analysis (GSEA) of ACE2-based gene expression patterns with the hallmark glycolysis gene set. (**D**) The expression profile of ACE2 across human cancers; data were derived from the TIMER database. (**E**) Kaplan-Meier curve analysis for overall survival in HCC patients (TCGA cohort) based on the median expression value of ACE2 expression; HR: hazard ratio. (**F**) Kaplan-Meier curve analysis for disease-free survival in HCC patients (TCGA cohort) based on the median expression value of ACE2 expression; HR: hazard ratio. (**G**) Immunofluorescence analysis showed ACE2 signals in human normal liver and HCC; scale bar, 50 μm. (**H**) Representative images of ACE2 expression in HCC tissues and corresponding normal liver tissues. “-” indicates no staining, “+” indicates weak staining, “++” indicates moderate staining, and “+++” indicates strong staining. “-” and “+” were defined as low ACE2 expression, while “++” and “+++” were defined as high ACE2 expression. (**I**) Kaplan-Meier curve analysis for overall survival in HCC patients based on the IHC results of ACE2 staining in Ren Ji cohort (n = 202). ACC, Adrenocortical carcinoma; BLCA, Bladder urothelial carcinoma; BRCA, Breast invasive carcinoma; CESC, Cervical squamous cell carcinoma and endocervical adenocarcinoma; CHOL, Cholangio carcinoma; COAD, Colon adenocarcinoma; DLBC, Lymphoid neoplasm diffuse large B-cell lymphoma; ESCA, Esophageal carcinoma; GBM, Glioblastoma multiforme; HNSC, Head and neck squamous cell carcinoma; KICH, Kidney chromophobe; KIRC, Kidney renal clear cell carcinoma; KIRP, Kidney renal papillary cell carcinoma; LAML, Acute myeloid leukemia; LGG, Brain lower grade glioma; LIHC, Liver hepatocellular carcinoma; LUAD, Lung adenocarcinoma; LUSC, Lung squamous cell carcinoma; MESO, Mesothelioma; OV, Ovarian serous cystadenocarcinoma; PAAD, Pancreatic adenocarcinoma; PCPG, Pheochromocytoma and paraganglioma; PRAD, Prostate adenocarcinoma; READ, Rectum adenocarcinoma; SARC, Sarcoma; SKCM, Skin cutaneous melanoma; STAD, Stomach adenocarcinoma; TGCT, Testicular germ cell tumors; THCA, Thyroid carcinoma; THYM, Thymoma; UCEC, Uterine corpus endometrial carcinoma; UCS, Uterine carcinosarcoma; UVM, Uveal melanoma. *P < 0.05; **P < 0.01; ***P < 0.001.

To decipher the potential negative regulators of HCC glycolysis, we leveraged the molecular profiles of Liver hepatocellular carcinoma (LIHC, n = 371) from the TCGA cohort (**Figure 1A**). Based on a 16-gene expression signature including genes specific to glycolysis, we divided the LIHC samples into two groups (glycolysis-high vs. glycolysis-low) and identified 605 differentially expressed protein-coding genes (DEGs) that negatively associated with glycolysis (**Supplementary Table 1**). Among these DEGs, several top hits (*SLC10A1*, *CYP3A4*, *SPP2*, and *LECT2*) were also revealed to be negative glycolytic regulators in HCC by other group 31, indicating that our analysis was built on the meaningful context of aerobic glycolysis. Moreover, 501 prognosis-associated genes and 721 differentially expressed and downregulated genes were found in HCC. By merging genes from the above three lists, we identified 8 candidates (*DNASE1L3*, *SLC22A1*, *ACE2*, *CES3*, *CCL14*, *GYS2*, *ADH4*, and *CFHR3*) (**Figure 1B**). Notably, DNASE1L3 has been reported to inhibit HCC progression by inducing cell apoptosis and weakening tumor glycolysis 32. SLC22A1 is downregulated in HCC and may affect the response to sorafenib 33. A preventive role of CES3 protein has been reported in the early stages of liver cancer development 34. CCL14 suppresses cell proliferation and promotes cell apoptosis in HCC 35. GYS2 functions as a tumor suppressor in HCC via regulation of p53 activity 36, and ADH4 serves as a prognostic marker in HCC 37. CFHR3 is a novel prognostic biomarker for HCC 38. Given the known roles of these seven candidates, we focused on ACE2, which is poorly studied in HCC. Based on the expression level of ACE2, we performed gene set enrichment analysis (GSEA) and the result revealed that ACE2 was significantly and inversely associated with the glycolysis gene signature (**Figure 1C**). Downregulation of ACE2 was noticed in several cancer types, including HCC (**Figure 1D**). Additionally, compared to patients with lower ACE2 expression, patients with higher ACE2 expression had significantly improved overall survival (HR = 0.54; 95% CI, 0.38-0.76; P = 4e-04) (**Figure 1E**) and disease-free survival (HR = 0.44; 95% CI, 0.28-0.68; P = 2e-04) (**Figure 1F**). Immunofluorescence analysis showed the positive staining of ACE2 was mostly membrane and cytoplasmic staining (**Figure 1G**). To further address the prognostic value of ACE2 in HCC, immunohistochemical analysis of ACE2 expression in an HCC tissue microarray (Ren Ji cohort) was performed. As a result, ACE2 expression was downregulated in HCC tissues compared with corresponding normal liver tissues (**Figure 1H**). Consistently, higher ACE2 expression predicted a better prognosis in HCC patients (**Figure 1I**). Collectively, these findings suggest that a close connection between ACE2 and aerobic glycolysis in HCC.

### ACE2 is a negative regulator of aerobic glycolysis in HCC

**Figure 2:**
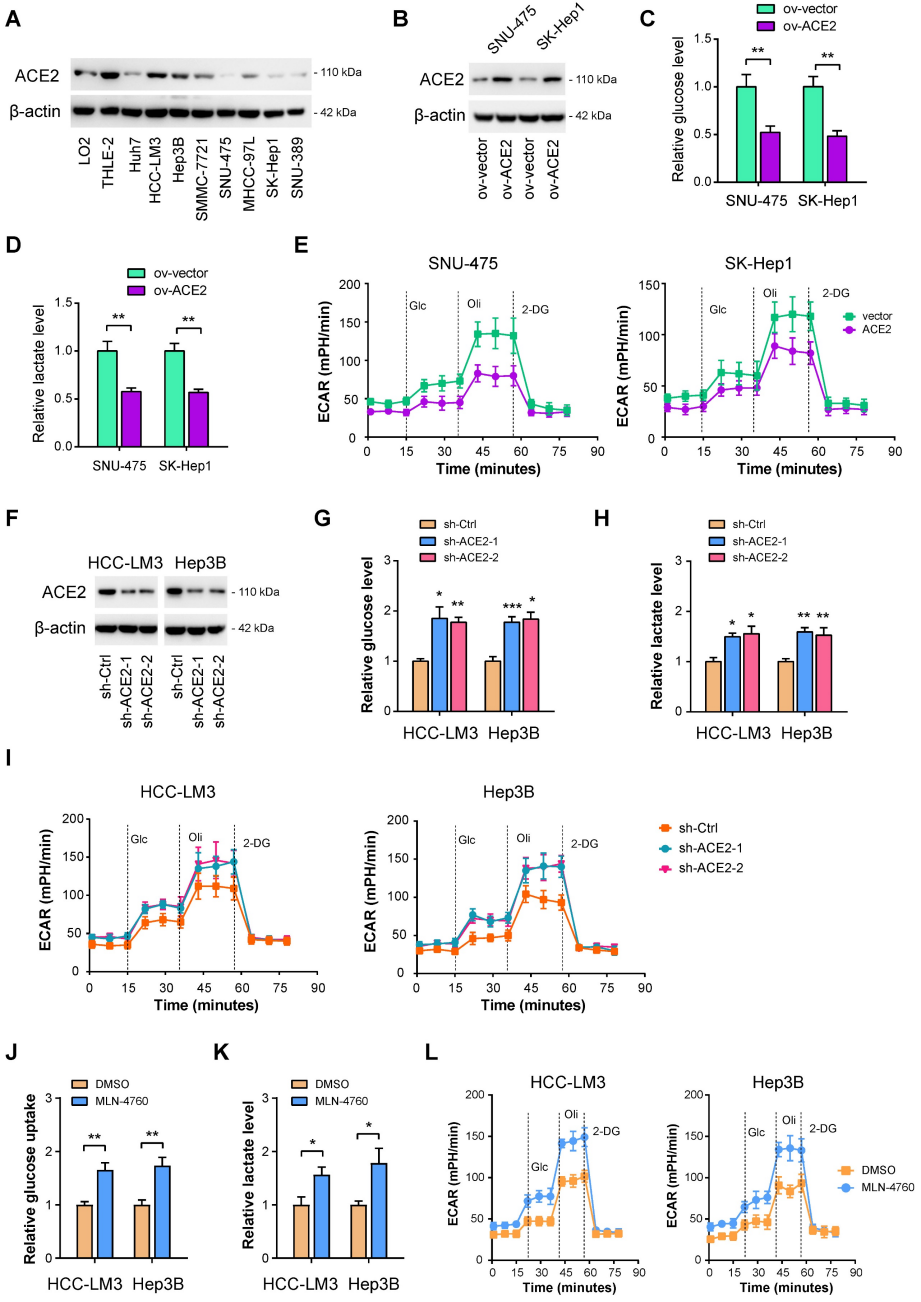
** ACE2 is a negative regulator of aerobic glycolysis in HCC.** (**A**) The protein levels of ACE2 in liver cancer cells and the nonmalignant cells (LO2 and THLE-2) were verified by Western blotting. (**B**) The overexpression efficiency of ACE2 in SNU-475 and SK-Hep1 cells was tested by Western blotting analysis. (**C-E**) The influences of ACE2 overexpression on the glucose uptake (**C**, n = 3), lactate release (**D**, n = 3), and extracellular acidification rate (**E**, n = 3) in SNU-475 and SK-Hep1 cells. (**F**) Two independent shRNAs against ACE2 were used for knockdown of ACE2 and the knockdown efficiency was verified by Western blotting in HCC-LM3 and Hep3B cells. (**G-I**) The influences of ACE2 knockdown on the glucose uptake (**G**, n = 3), lactate release (**H**, n = 3), and extracellular acidification rate (**I**, n = 3) in HCC-LM3 and Hep3B cells. (**J-L**) The influences of ACE2 inhibitor MLN-4760 (5 μM) on the glucose uptake (**J**, n = 3), lactate release (**K**, n = 3), and extracellular acidification rate (**L**, n = 3) in HCC-LM3 and Hep3B cells. *P < 0.05; **P < 0.01; ***P < 0.001.

To investigate whether ACE2 can inhibit HCC glycolysis or not, both gain-of-function and loss-of-function experiments were performed. Firstly, Western blotting was carried out to evaluate the protein level of ACE2 in HCC cell lines. Compared with the nonmalignant LO2 and THLE-2 cell lines, ACE2 was less expressed in HCC cell lines (**Figure 2A**). Then, two cell lines, SNU-475 and SK-Hep1, with lower ACE2 expression, were selected for the gain-of-function study (**Figure 2B**). It is worth mentioning that SK-Hep-1 is a cell line isolated from the ascitic fluid of a patient with liver adenocarcinoma and has been identified as being of endothelial origin 39. To measure the changes in the glycolytic flux, we detected glucose uptake, lactate release, extracellular acidification rate (ECAR), and the mRNA level of glycolytic genes after ACE2 overexpression. In SNU-475 and SK-Hep1 cells, ACE2 overexpression led to a remarkable reduction in glucose uptake (**Figure 2C**), lactate release (**Figure 2D**), ECAR (**Figure 2E**), and expression of glucose transporter (SLC2A1) and glycolytic genes (SLC2A1, HK2, ENO1, PFKL, LDHA, and PDK1) (**Supplementary Figure 1A**). Moreover, loss-of-function experiments were done in HCC-LM3 and Hep3B cells. Two shRNAs against ACE2 resulted in marked downregulation in the ACE2 protein level (**Figure 2F**). In contrast to ACE2 overexpression, ACE2 knockdown promoted glucose uptake (**Figure 2G**), lactate release (**Figure 2H**), ECAR (**Figure 2I**), and expression of glycolytic components (**Supplementary Figure 1B**) in HCC-LM3 and Hep3B cells. Moreover, inhibition of ACE2 with 5 μM MLN-4760 phenocopied the glycolysis-promoting effects of ACE2 knockdown in HCC cells (**Figure 2J-L**). To rule out the decrease of glucose metabolism may be a direct effect from impairing cellular growth, we further checked the glucose uptake and lactate production at 12 h, at which time cellular growth remained unaffected. As a result, the effects of ACE2 on HCC glucose metabolism were still existed (**Supplementary Figure 2**). Taken together, ACE2 is evidently involved in the regulation of glycolytic metabolism.

### ACE2 depends on the Ang-(1-7)/Mas receptor axis to inhibit aerobic glycolysis

**Figure 3:**
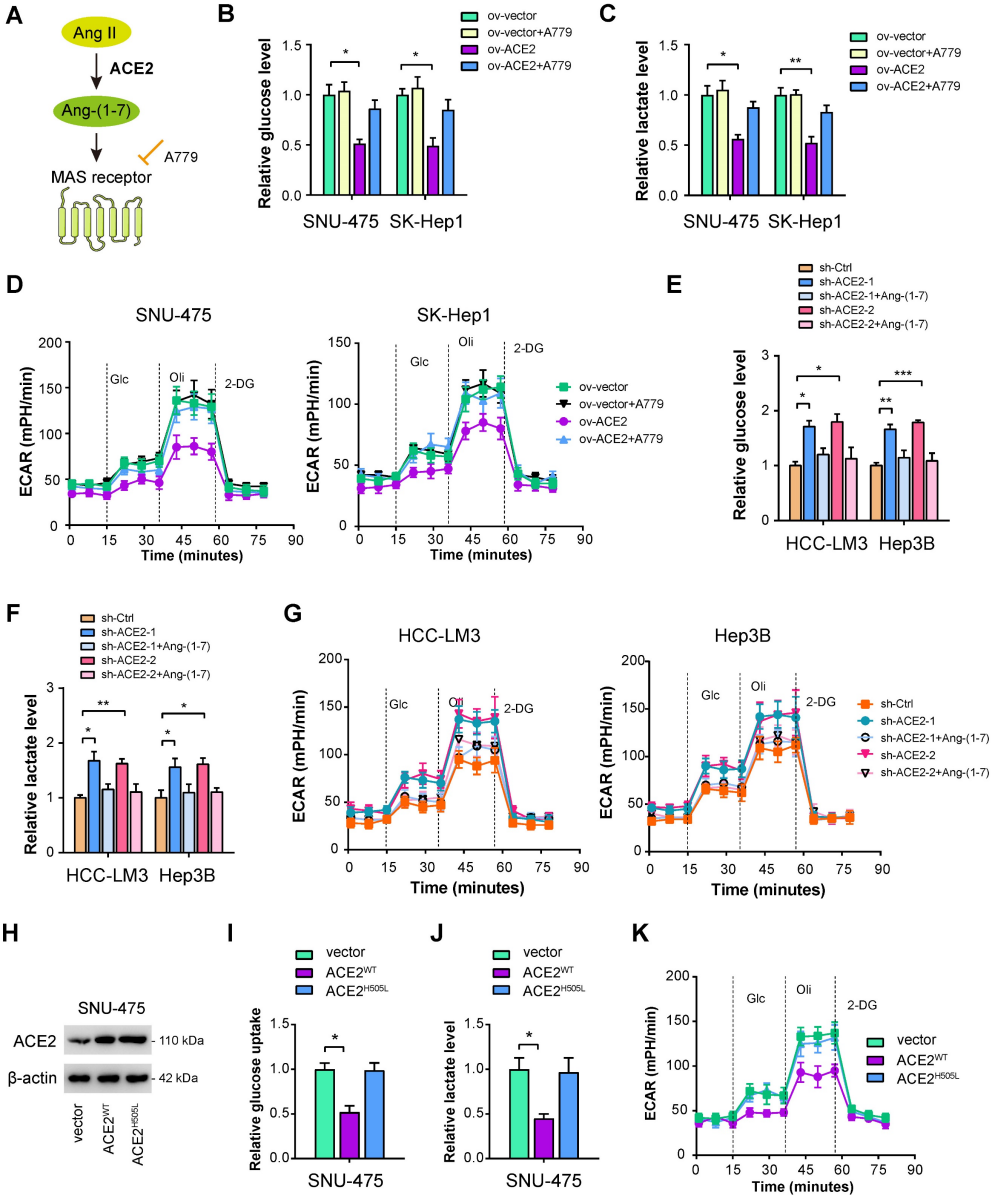
** ACE2 depends on the Ang-(1-7)/Mas receptor axis to inhibit aerobic glycolysis.** (**A**) Simplified view of the ACE2/Ang-(1-7)/Mas receptor axis. (**B-D**) The effects of ACE2 overexpression on the glucose uptake (**B**, n = 3), lactate release (**C**, n = 3), and extracellular acidification rate (**D**, n = 3) in the presence or absence of A779 (1 μM) were measured in SNU-475 and SK-Hep1 cells. (**E-G**) The influences of ACE2 knockdown on the glucose uptake (**E**, n = 3), lactate release (**F**, n = 3), and extracellular acidification rate (**G**, n = 3) in the presence or absence of Ang-(1-7) (10^-8^ M) were detected in HCC-LM3 and Hep3B cells. (**H**) The overexpression efficiency of wild type ACE2 (ACE2^WT^) and enzymatic-dead ACE2 (ACE2^H505L^) in SNU-475 cells was tested by Western blotting analysis. (**I-K**) The influences of ACE2^WT^ and ACE2^H505L^ on the glucose uptake (**I**, n = 3), lactate release (**J**, n = 3), and extracellular acidification rate (**K**, n = 3) in SNU-475 cells. *P < 0.05; **P < 0.01; ***P < 0.001.

ACE2 cleaves Ang II to generate Ang-(1-7), which further activates Mas receptor to initiate a downstream signaling cascade (**Figure 3A**). To determine whether this is the case, we verified the roles of ACE2 on HCC glycolysis in the presence or absence of Ang-(1-7) and the Mas receptor inhibitor A779 40. As displayed in **Figure 3B-D**, the addition of 1 μM A779 restored the decrease in glucose uptake, lactate release, and ECAR induced by ACE2 overexpression. Moreover, we added Ang-(1-7) (10^-8^ M) to the culture medium of ACE2 knockdown cells. Expectedly, increased glycolytic capacity observed in ACE2 knockdown cells was largely compromised by Ang-(1-7) treatment as evidenced by the reduced level of glucose uptake, lactate release, and ECAR (**Figure 3E-G**). To further test whether enzymatic activity of ACE2 is required for its regulatory role in glycolysis, we transfected SNU-475 cells with ACE2 catalytic site histidine mutation (H505L) (**Figure 3H**). Compared with wild type ACE2, enzymatic-dead ACE2 failed to suppress glycolysis as revealed by glucose uptake, lactate release, and ECAR (**Figure 3I-K**). Taken together, the enzymatic activity of ACE2 and Mas receptor are needed for the inhibitory roles of ACE2 on HCC glycolysis.

### ACE2 suppresses HIF1α activity in HCC

**Figure 4:**
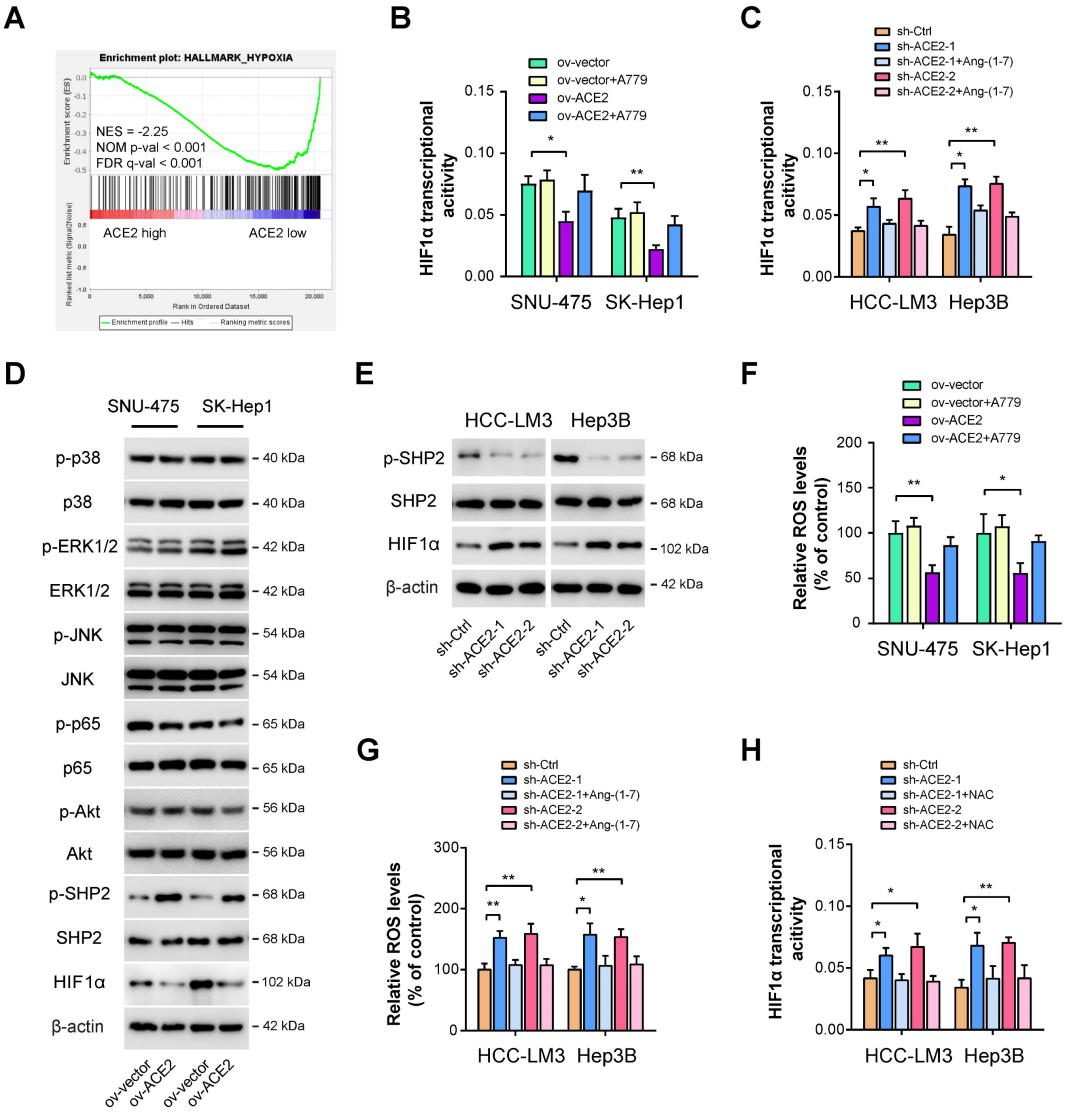
** ACE2 suppresses HIF1α activity in HCC.** (**A**) GSEA plot showed a close link between ACE2 expression and hypoxia in HCC samples from the TCGA cohort. (**B**) The effects of ACE2 overexpression on HIF1α transcriptional activity in the presence or absence of A779 (1 μM) were measured in SNU-475 and SK-Hep1 cells. (**C**) The effects of ACE2 knockdown on HIF1α transcriptional activity in the presence or absence of Ang-(1-7) (10^-8^ M) were measured in HCC-LM3 and Hep3B cells. (**D**) The effects of ACE2 overexpression on the activity of ACE2-associated signaling pathways (p38 MAPK, ERK1/2, JNK, NF-κB, AKT, SHP2, and HIF1α) in SNU-475 and SK-Hep1 cells was analyzed by Western blotting. (**E**) The levels of p-SHP2, SHP2, and HIF1α in sh-Ctrl and sh-ACE2 HCC-LM3 and Hep3B cells was analyzed by Western blotting. (**F**) The effects of ACE2 overexpression on ROS generation in the presence or absence of A779 (1 μM) were measured in SNU-475 and SK-Hep1 cells. (**G**) The effects of ACE2 knockdown on ROS generation in the presence or absence of Ang-(1-7) (10^-8^ M) were measured in HCC-LM3 and Hep3B cells. (**H**) The effects of ACE2 knockdown on HIF1α transcriptional activity in the presence or absence of 1 mM N-acetyl cysteine (NAC) treatment were measured in HCC-LM3 and Hep3B cells. *P < 0.05 and **P < 0.01.

Next, we aimed to delineate the underlying molecular mechanism responsible for ACE2-mediated glycolytic changes. As analyzed above, we also identified other molecular differences in the molecular profile data associated with ACE2 expression. As a result, significant enrichment in hypoxia signaling was observed (**Figure 4A**). Given that HIF1α is a key transcriptional factor for glycolytic metabolism 14, we, therefore, investigated the link between ACE2 and HIF1α. Using a commercial detection kit, we first detected HIF1α transcriptional activity upon ACE2 overexpression. Intriguingly, HIF1α transcriptional activity was markedly attenuated by ACE2 overexpression and can be further rescued by the addition of A779 (**Figure 4B**). In contrast, ACE2 knockdown increased HIF1α transcriptional activity, which can also be blocked by the addition of Ang-(1-7) (**Figure 4C**). Previously, several known downstream signaling molecules of Mas receptor have been revealed, such as MAPKs (p38, ERK1/2, JNK), NF-κB, and AKT 41. Moreover, Ang-(1-7) can counterbalance Ang II signaling via hijacking Ang II-induced SHP-2 dephosphorylation and reactive oxygen species (ROS) generation 42. By Western blotting analysis, we observed that ACE2 overexpression significantly increased the phosphorylated level of SHP2 and decreased HIF1α activity, but had no significant implications on MAPKs, NF-κB, and AKT signaling in SNU-475 and SK-Hep1 cells (**Figure 4D**). Conversely, ACE2 knockdown suppressed SHP2 phosphorylation and increased the HIF1α level (**Figure 4E**), indicating that ACE2 might modulate SHP2 phosphorylation to influence the HIF1α level. To further determine whether ROS is essential for ACE2-dependent HIF1α activity, we detected ROS levels upon manipulation of the ACE2/Ang-(1-7)/Mas receptor. As shown in **Figure 4F**, ACE2 overexpression reduced ROS level, and inhibition of Mas receptor with A779 rescued ROS level. In opposite, ACE2 knockdown increased ROS level, and the addition of Ang-(1-7) further blocked ROS generation (**Figure 4G**). To elucidate whether ROS is responsible for ACE2-dependent HIF1α activity in HCC, we blocked ROS function with the addition of the antioxidant N-acetylcysteine (NAC). Indeed, increased HIF1α transcriptional activity induced by ACE2 knockdown was blocked by NAC (**Figure 4H**). Likewise, NAC also blocked the increased glucose uptake, lactate release, and ECAR induced by ACE2 knockdown in HCC-LM3 and Hep3B cells (**Supplementary Figure 3**). Collectively, ACE2 may inhibit ROS generation to regulate HIF1α activity and aerobic glycolysis in HCC.

### ACE2 overexpression inhibits HCC tumor growth

**Figure 5:**
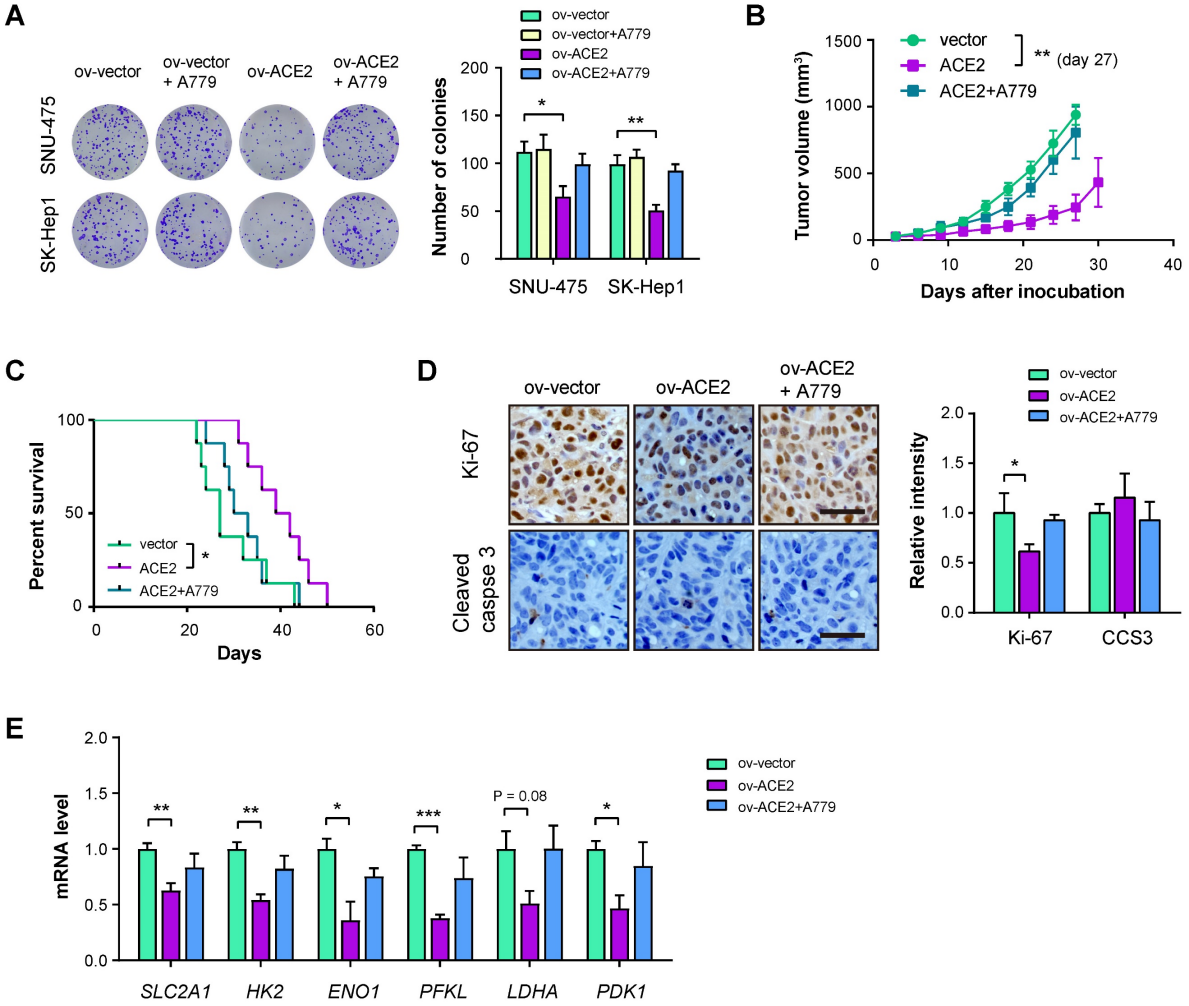
** ACE2 overexpression inhibits HCC tumor growth.** (**A**) The effects of ACE2 overexpression on *in vitro* proliferation of SNU-475 and SK-Hep1 cells in the presence or absence of A779 (1 μM) were measured by colony formation assay (n = 3). (**B**) ov-vector and ov-ACE2 SK-Hep1 cells were subcutaneously injected into the nude mice (n = 5 per group) and tumor growth was monitored with or without A779 treatment. (**C**) Survival curve of mice in the ov-vector, ov-ACE2, and ov-ACE2 + A779 groups (n = 8 per group). (**D**) IHC analysis of Ki-67 and cleaved Caspase-3 (CCS3) in the tumor tissues from the ov-vector, ov-ACE2, and ov-ACE2 + A779 groups. Scale bar, 50 μm. (**E**) qRT-PCR analysis of glycolytic components (SLC2A1, HK2, ENO1, PFKL, LDHA, and PDK1) in the tumor tissues from the ov-vector, ov-ACE2, and ov-ACE2 + A779 groups. Scale bar, 50 μm. *P < 0.05; **P < 0.01; ***P < 0.001.

To answer whether ACE2 plays tumor-suppressive roles in HCC, we performed *in vitro* and *in vivo* experiments. Plate colony formation assay showed that ACE2 overexpression reduced *in vitro* cell proliferation of SNU-475 and SK-Hep1 cells and the inhibitory roles of ACE2 on cell proliferation can be restored by A779 (**Figure 5A**). Furthermore, we generated a subcutaneous xenograft model by implanting SK-Hep1 cells into the flanks of immunocompromised mice. The result showed that ACE2 overexpression retarded tumor growth, while A779 blocked the effect of ACE2 overexpression (**Figure 5B**). In another cohort of animal experiments, mice in the ov-ACE2 group had improved survival compared with mice in the ov-vector or ov-ACE2 + A779 group (**Figure 5C**). The inhibitory effect of the ACE2/Mas receptor axis was further supported by IHC analysis of the proliferation index Ki-67. Notably, cell apoptosis was not induced by genetic manipulation of ACE2, either overexpression or knockdown, as demonstrated by the IHC staining of cleaved caspase 3 (CCS3) and *in vitro* caspase-3/7 activity (**Figure 5D** and **Supplementary Figure 4**). Real-time qPCR analysis of glucose transporter and glycolytic genes in the xenograft tumor tissues showed that ACE2 overexpression suppressed the mRNA levels of SLC2A1, HK2, ENO1, PFKL, LDHA, and PDK1, while inhibition of Mas receptor with A779 restored the expression of glycolytic components (**Figure 5E**).

### ACE2 knockdown promotes HCC tumor growth

**Figure 6:**
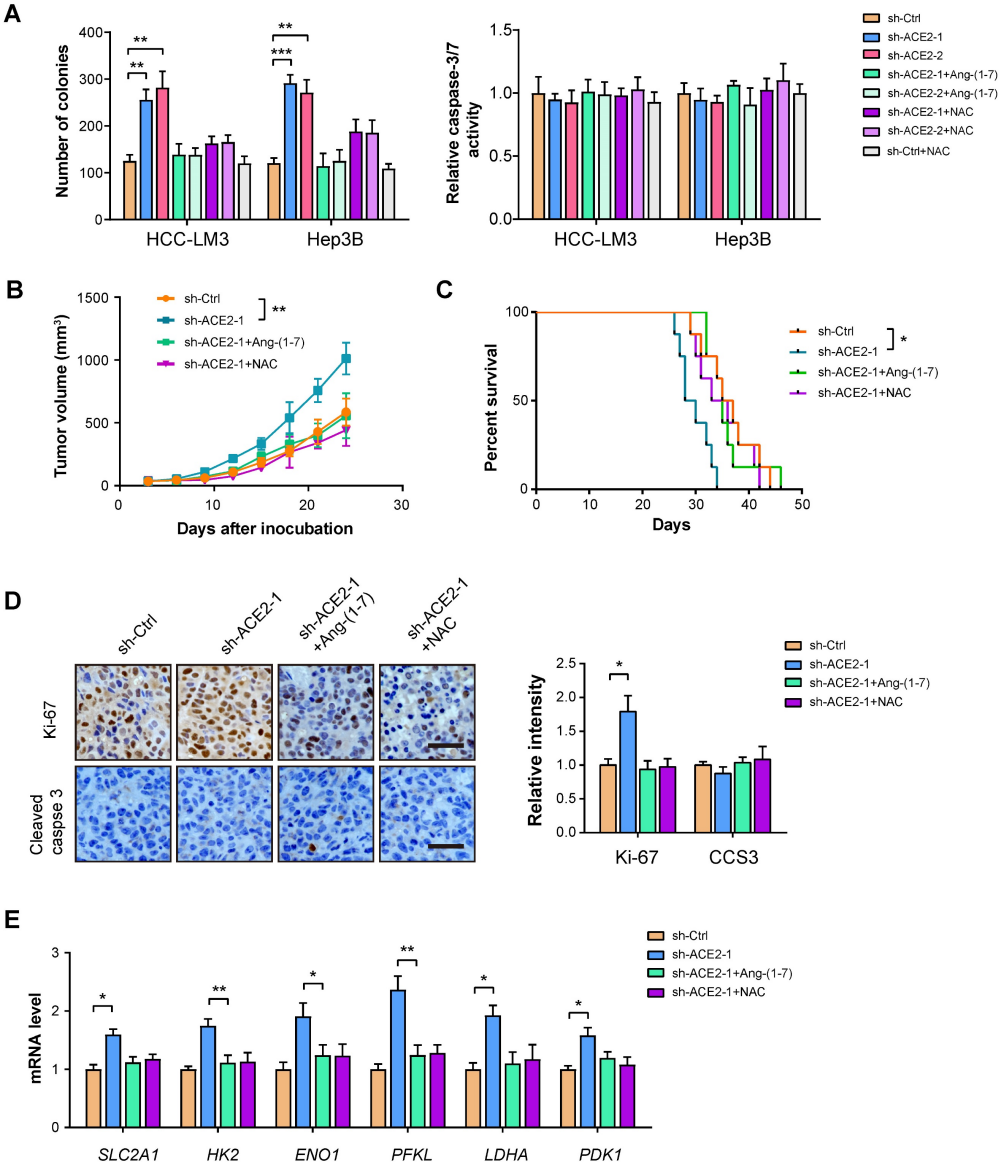
** ACE2 knockdown promotes HCC tumor growth.** (**A**) The effects of ACE2 knockdown on *in vitro* proliferation and apoptosis of HCC-LM3 and Hep3B cells in the presence or absence of Ang-(1-7) (10^-8^ M) or NAC treatment (1 mM) were measured by colony formation assay and caspase-3/7 activity (n = 3), respectively. (**B**) sh-Ctrl and sh-ACE2 HCC-LM3 cells were subcutaneously injected into the nude mice (n = 5 per group) and tumor growth was monitored with or without Ang-(1-7) or NAC treatment. (**C**) Survival curve of mice in the sh-Ctrl, sh-ACE2, sh-ACE2 + Ang-(1-7), and sh-ACE2 + NAC groups (n = 8 per group). (**D**) IHC analysis of Ki-67 and cleaved Caspase-3 (CCS3) in the tumor tissues from the sh-Ctrl, sh-ACE2, sh-ACE2 + Ang-(1-7), and sh-ACE2 + NAC groups; Scale bar, 50 μm. (**E**) qRT-PCR analysis of glycolytic components (SLC2A1, HK2, ENO1, PFKL, LDHA, and PDK1) in the tumor tissues from the sh-Ctrl, sh-ACE2, sh-ACE2 + Ang-(1-7), and sh-ACE2 + NAC groups. *P < 0.05; **P < 0.01; ***P < 0.001.

To further strengthen the inhibitory effect of ACE2 on tumor growth, we performed a loss-of-function experiment in HCC-LM3 and Hep3B cells. Plate colony formation showed that ACE2 knockdown promoted the *in vitro* proliferation of HCC-LM3 and Hep3B cells, but had no significant effects on cell apoptosis as indicated by Caspase-3/7 activity (**Figure 6A**). *In vivo* studies showed that tumors formed by sh-ACE2-1 HCC-LM3 cells grew more rapidly than tumors formed by sh-Ctrl HCC-LM3 cells (**Figure 6B**). Mice in the sh-ACE2-1 group had a lesser survival time compared with mice in the sh-Ctrl group (**Figure 6C**). The *in vivo* growth-promoting effects of ACE2 knockdown were also supported by Ki-67 staining (**Figure 6D**). Moreover, ACE2 knockdown increased the expression of glycolytic genes in xenograft tumor tissues (**Figure 6E**). Notably, both Ang-(1-7) and NAC can compromise the effects of ACE2 knockdown on tumor growth and the expression of glycolytic components (**Figure 6A-E**). Notably, hijacking tumor glycolytic metabolism with the glycolysis inhibitor 2-DG largely abrogated the growth-promoting effects induced by ACE2 knockdown (**Supplementary Figure 5**). Collectively, these findings above indicate that ACE2 acts as a tumor suppressor in HCC.

### Clinical relevance of ACE2, p-SHP2, and HIF1α in HCC samples

**Figure 7:**
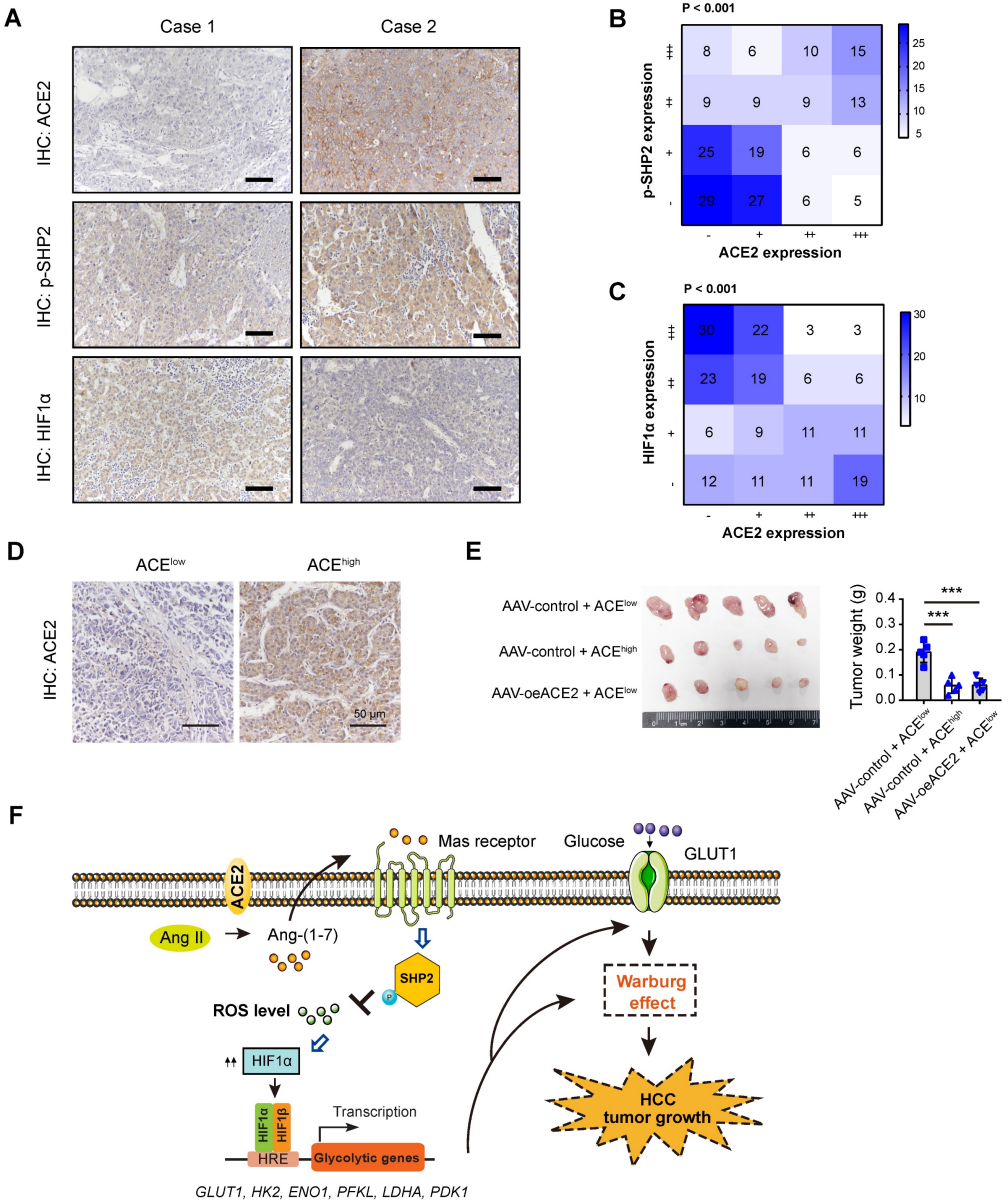
** Clinical relevance of ACE2 in HCC samples**. (**A**) Representative IHC staining of ACE2, HIF1α or p-SHP2 protein levels in human HCC specimens (n = 202); scale bar, 50 μm. (**B**) Correlation between ACE2 and p-SHP2 protein was determined by Spearman analysis. (**C**) Correlation between ACE2 and HIF1αprotein was determined by Spearman analysis. (**D**) For PDX model, representative IHC staining of ACE2 in two HCC samples was shown. (**E**) The effect of ACE2 on the tumor growth of PDX xenograft was studied by a subcutaneous xenograft model. (**F**) The schematic diagram for the molecular mechanism of ACE2-mediated suppression of HIF1α activity and tumor growth in HCC. In brief, ACE2 metabolizes Ang II to Ang-(1-7), which activates Mas receptor and leads to the phosphorylation of SHP2. SHP2 activation blocks ROS generation, which further stabilizes HIF1α protein. HIF1α acts as a key transcriptional factor to induce the expression of glucose transporters and glycolytic genes and enhances the Warburg effect. Finally, tumor growth of HCC is suppressed by ACE2 due to compromised glycolytic flux. ***P < 0.001.

To add the clinical relevance, we first analyzed the protein expression of ACE2, phosphorylated-SHP2 (p-SHP2), and HIF1α by IHC in a cohort of 202 HCC patients. Representative IHC images for ACE2, p-SHP2, and HIF1α were shown in **Figure 7A**. As a result, a close and positive correlation between ACE2 and p-SHP2 was revealed (**Figure 7B**). In contrast, ACE2 expression was negatively associated with HIF1α intensity (P < 0.001) in the HCC samples (**Figure 7C**). To test the therapeutic value of ACE2, we generated a patient-derived xenograft (PDX) model with two HCC samples, one sample with low ACE expression and one sample with high ACE2 expression (**Figure 7D**). As a result, xenografts from ACE2^low^ tumors grew faster than ACE2^high^ tumors. Interestingly, intratumoral injection of AAV-ovACE2 blocked the expression of ACE2^low^ tumors (**Figure 7E**). Therefore, these results further confirm the ACE2-mediated molecular mechanism in the clinical setting.

## Discussion

Activation of oncogenes and inactivation of tumor suppressors are essential for the initiation and progression of HCC 9. Ample evidence has deciphered the roles of oncogene-mediated metabolic reprogramming, however, limited knowledge is known about the underlying functional suppressor of aerobic glycolysis in HCC. In this study, we leveraged the molecular profile of HCC from the TCGA cohort and identified a series of DEGs that were negatively associated with aerobic glycolysis. Among them, ACE2 was demonstrated to exert its antitumor effect against HCC via generation of Ang-(1-7), which further acts on the G protein-coupled receptor Mas. Subsequently, pathway analysis revealed that p-SHP2/ROS/HIF1α signaling was the downstream cascade of the ACE2/Ang-(1-7)/Mas receptor axis (**Figure 7F**).

In contrast to the actions of the ACE/Ang II/AT1 receptor, the ACE2/Ang-(1-7)/Mas receptor axis plays a counter-regulatory role on the same target, such as myocardium, blood vessels, brain, kidney, and other organs 43, 44. In tumors, both positive and negative roles of ACE2 have been reported 23. As shown in **Figure 1**, ACE2 expression was downregulated in most cancer types (BRCA, KICH, LIHC, PCPG, PRAD, and THCA), and upregulated ACE2 expression was observed at CESC, ESCA, KIRP, LUAD, and UCEC. For the expression pattern of ACE2 in liver tissues, Hikmet et al. performed IHC analysis of ACE2 in a tissue microarray containing 18 cases of liver samples and showed that ACE2 was very low in hepatocytes 45. In contrast, other studies revealed that ACE2 signals are expressed on hepatocytes, bile duct cells and liver endothelial cells 46. In contrast, we revealed that ACE2 was variedly expressed in non-tumor liver tissues though a large-scale sample investigation (n = 202). This discrepancy might be affected by multiple factors including but not limited to different cohort, the antibodies used and sample size detected. The role of ACE2 in cancers might be cancer-specific. Indeed, ACE2/Ang-(1-7)/Mas receptor has inhibitory effects on cancer cell proliferation in breast cancer, prostate cancer, and lung cancer 26, 47, 48, while Ang-(1-7) promotes cancer cell migration and invasion in human renal cell carcinoma 49. In most cases, ACE2 is tumor-suppressive and acts as an inhibitor of tumor growth, metastasis, and angiogenesis. In alignment with previous report, we revealed that ACE2 is downregulated in HCC and higher ACE2 expression is associated with a better prognosis in HCC patients. Interestingly, we for the first time presented a previous unprecedented role of ACE2 in regulating aerobic glycolysis in HCC. Both *in vitro* and in vivo gain-of-function and loss-of-function studies supported the tumor suppressor function on aerobic glycolysis. Different from the situation in HCC, the ACE2/Ang-(1-7)/Mas receptor axis can enhance glucose uptake by skeletal muscle and inhibit hepatic gluconeogenesis 50, suggesting the context-dependent roles of ACE2.

The ACE2/Ang-(1-7)/Mas receptor can couple many intracellular signaling pathways to influence a range of actions under both physiology and disease stages 40. In breast cancer, ACE2/Ang-(1-7)/Mas receptor axis inhibits the sore-operated calcium entry and PAK1/NF-κB/Snail1 pathway 51. However, we failed to notice significant changes in the NF-κB signal upon ACE2 overexpression in two HCC cell lines. ACE2/Ang-(1-7)/Mas receptor axis is also involved in the inhibition of MAPK signaling in diverse situations, such as inflammation and cancers 20, 41. Interestingly, p-P38, p-ERK1/2, and p-JNK remained largely unaltered after ACE2 overexpression. Through pathway analyses and functional verification, we identified HIF1α as a key change in response to ACE2 knockdown or overexpression.

In line with our previous findings 18, intracellular ROS contributed to HIF1α protein stabilization in HCC as blocking ROS with NAC suppressed HIF1α activity. In human endothelial cells, Ang-(1-7) can counterregulate Ang II/AT1 receptor signaling to reduce ROS generation via the phosphorylation of SHP2 42. Consistently, genetic manipulation of ACE2 led to significant changes in the phosphorylated level of SHP2 in HCC cells. Therefore, we provided new insight regarding the molecular mechanism underlying the inhibitory role of the ACE2/Ang-(1-7)/Mas receptor axis in human cancers. Up to date, accumulating ACE2 agonists have been designed for many conditions, such as hypertension, myocardial Ischemia, type 2 diabetes mellitus, bone cancer, chondrosarcoma, and clear cell sarcoma of the kidney 27. For instance, the antitrypanosomal agent diminazene aceturate (DIZE), an activator for ACE2, has been reported to play beneficial effects in several clinical models of hypertension, myocardial infarction, type 1 diabetes and atherosclerosis 52. However, whether ACE2 activation are beneficial to cancer patients warrants further investigations. Since ACE inhibitors is widely used for cardiovascular diseases, our findings suggest that clinical use of ACE inhibitors may increase the incidence or the progression of HCC due to their potential roles in enhancing tumor glucose metabolism.

## Conclusions

Together, we identified ACE2 as a negative regulator against HCC glycolysis. Based on the background of aerobic glycolysis, activation of the ACE2/Ang-(1-7)/Mas receptor axis significantly retards tumor growth in HCC. Given that aberrant glucose metabolism is a general phenomenon in human solid cancers, targeting the ACE2/Ang-(1-7)/Mas receptor axis could be extended to other cancers. However, there are also several limitations in the present study. Firstly, effective assay is not developed to measure ACE2 activity in HCC cells and tumor tissues. Secondly, most of the data are acquired from *in vitro* cell experiments and *in vivo* nude mice, the implications of the ACE2/Ang-(1-7)/Mas receptor axis on the immune system are not studied.

## Supplementary Material

Supplementary figures and table.

## Abbreviations

HCC: hepatocellular carcinoma
ROS: reactive oxygen species
HIF1α: hypoxia-inducible factor 1α
ECAR: extracellular acidification rate
NAC: N-acetylcysteine
ACE2: angiotensin converting enzyme 2
GSEA: gene set enrichment analysis
TCA: tricarboxylic acid cycle
ATP: adenosine triphosphate
TME: tumor microenvironment
GLUT1: glucose transporter 1
HK2: hexokinase 2
PFKL: liver-type phosphofructokinase
LDHA: lactate dehydrogenase A
PKM2: pyruvate kinase M2
ccRCC: clear cell renal cell carcinoma
SARS-CoV-2: severe acute respiratory syndrome coronavirus 2
FDR: false discovery rate
BSA: bovine serum albumin
qRT-PCR: quantitative real-time PCR
LIHC: liver hepatocellular carcinoma
