# Supplementary material for: ACE2 negatively regulates the Warburg effect and suppresses hepatocellular carcinoma progression via reducing ROS-HIF1α activity
Source: Int J Biol Sci. 2023 May 11;19(8):2613–29. doi: 10.7150/ijbs.81498 (PMC10197896; doi:10.7150/ijbs.81498)
Supplement: Supplementary file 1 — Supplementary figures and table. [file ijbsv19p2613s1.pdf]

Supplementary Materials

Supplementary Figures

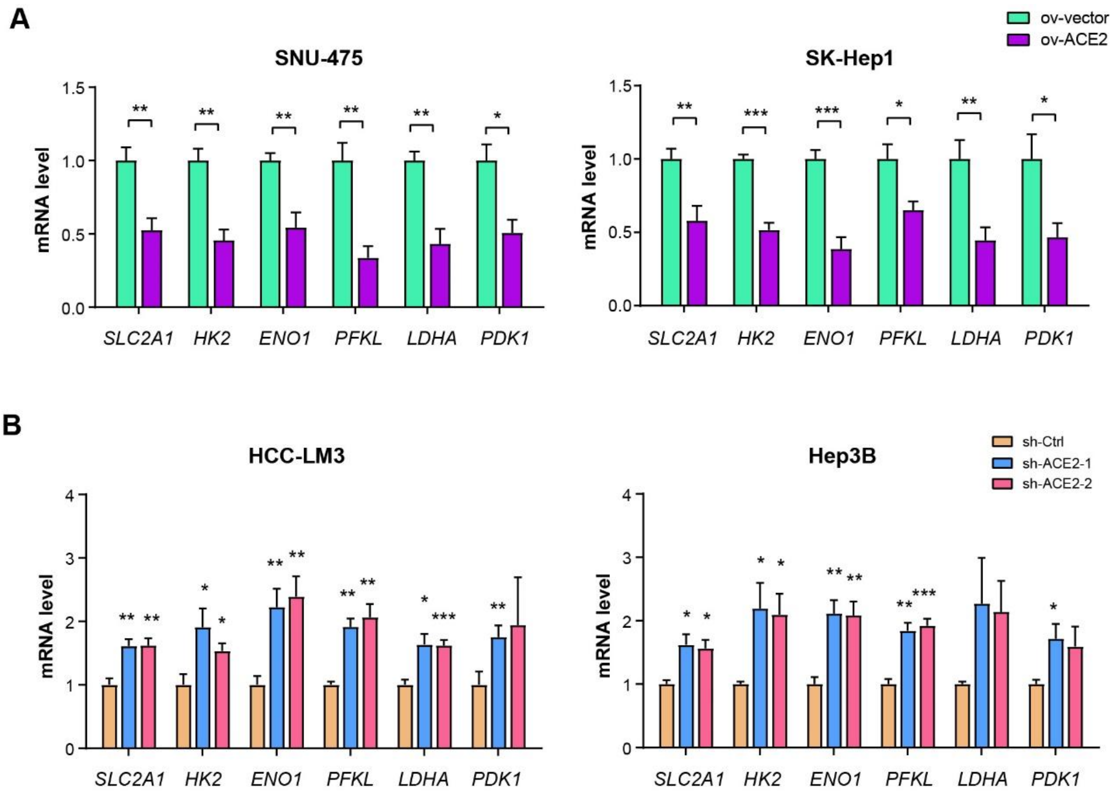

Supplementary Figure 1. Effects of ACE2 overexpression or knockdown on the expression of glycolytic components

(**A**) qRT-PCR analysis of glucose transporter (SLC2A1) and glycolytic genes (SLC2A1, HK2, ENO1, PFKL, LDHA, and PDK1) in the ov-vector and ov-ACE2 SUN-475 and SK-Hep1 cells (n = 3). (**B**) qRT-PCR analysis of glucose transporter (SLC2A1) and glycolytic genes (SLC2A1, HK2, ENO1, PFKL, LDHA, and PDK1) in the sh-Ctrl and sh-ACE2 HCC-LM3 and Hep3B cells (n = 3). *P < 0.05; **P < 0.01; ***P < 0.001.

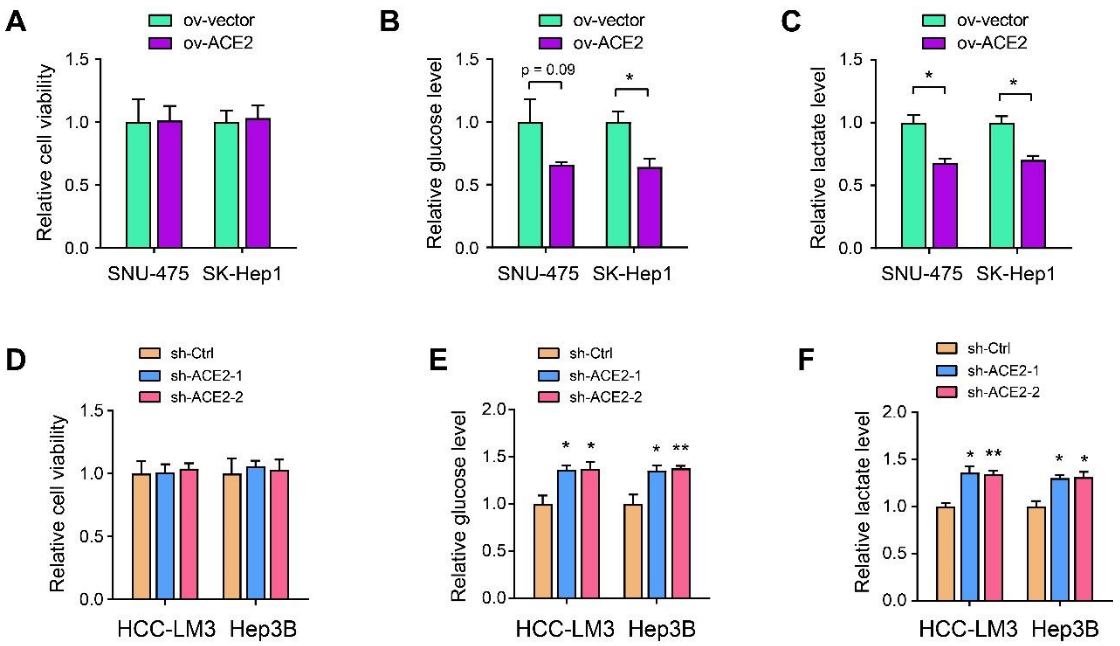

Supplementary Figure 2. ACE2 inhibits aerobic glycolysis in HCC cells. (**A**) CCK-8 assay showed the impacts of ACE2 overexpression on the cell proliferation of SNU-475 and SK-Hep1 cells at 12 h. (**B**) The influences of ACE2 overexpression on the glucose uptake in SNU-475 and SK-Hep1 cells at 12 h (n = 3). (**C**) The influences of ACE2 overexpression on the lactate release in SNU-475 and SK-Hep1 cells at 12 h (n = 3). (**D**) CCK-8 assay showed the impacts of ACE2 knockdown on the cell proliferation of HCC-LM3 and Hep3B cells at 12 h. (**E**) The influences of ACE2 knockdown on the glucose uptake in HCC-LM3 and Hep3B cells at 12 h (n = 3). (**F**) The influences of ACE2 knockdown on the lactate release in HCC-LM3 and Hep3B cells at 12 h (n = 3). *P < 0.05 and **P < 0.01.

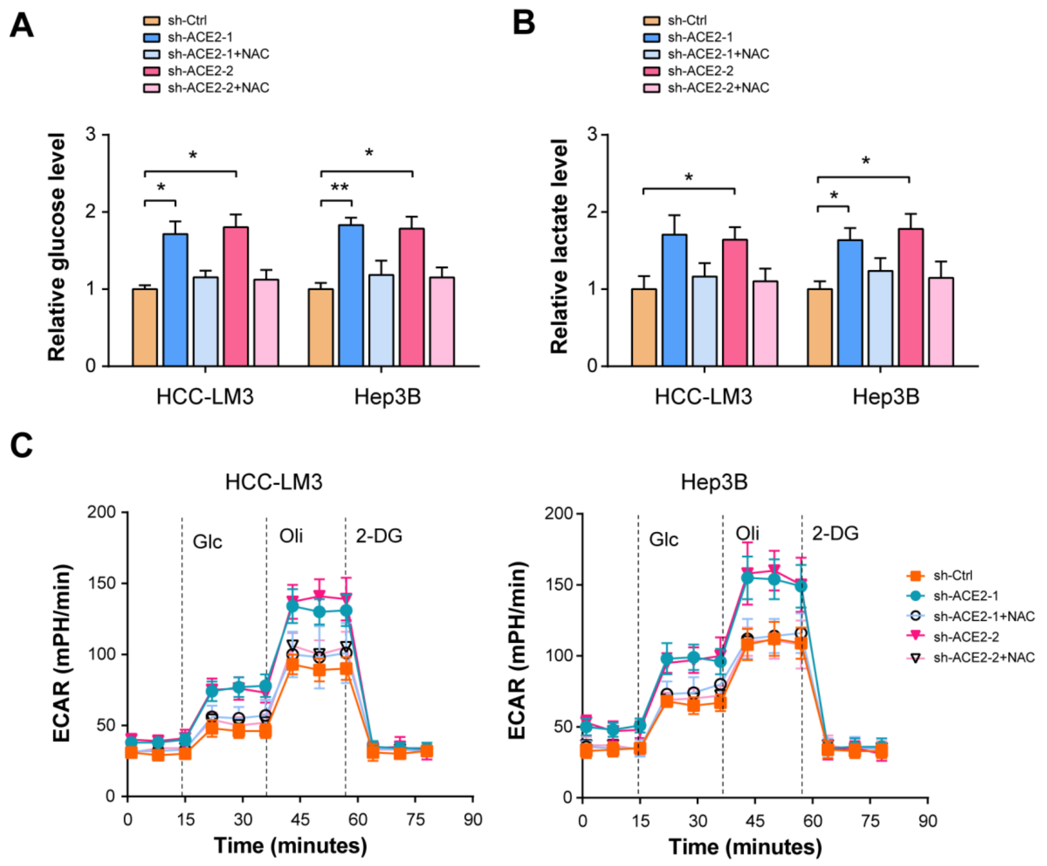

Supplementary Figure 3. NAC blocks enhanced aerobic glycolysis induced by ACE2 knockdown

(**A**) The effects of ACE2 knockdown on glucose uptake in the presence or absence of 1 mM N-acetyl cysteine (NAC) treatment were measured in HCC-LM3 and Hep3B cells. (**B**) The effects of ACE2 knockdown on lactate release in the presence or absence of 1 mM N-acetyl cysteine (NAC) treatment were measured in HCC-LM3 and Hep3B cells. (**C**) The effects of ACE2 knockdown on extracellular acidification rate in the presence or absence of 1 mM N-acetyl cysteine (NAC) treatment were measured in HCC-LM3 and Hep3B cells. *$P < 0.05$; **$P < 0.01$.

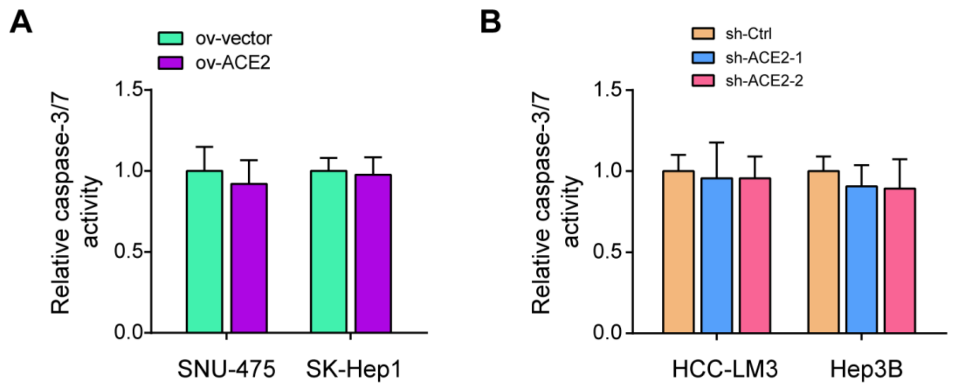

Supplementary Figure 4. ACE2 does not effect on HCC cell apoptosis

(**A**) Comparison of caspase-3/7 activity in the ov-vector and ov-ACE2 SUN-475 and SK-Hep1 cells (n = 3). (**B**) Comparison of caspase-3/7 activity in the sh-Ctrl and sh-ACE2 HCC-LM3 and Hep3B cells (n = 3).

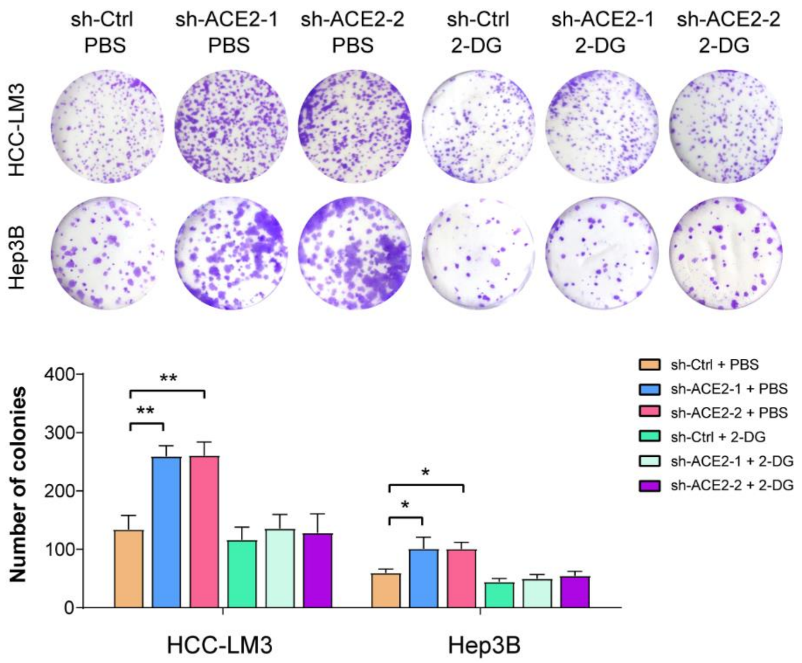

Supplementary Figure 5. ACE2 knockdown-mediated growth-promoting effects are glycolysis dependent. The effects of ACE2 knockdown on *in vitro* proliferation of HCC-LM3 and Hep3B cells in the presence or absence of 2-DG treatment were measured by colony formation assay (n = 3). *P < 0.05; **P < 0.01.

Supplementary Table 1. Genes negatively associated with HCC glycolysis

Gene symbol	Pvalue	adjPvalue	log2FC	Low	High
PAGE4	0.000334	0.001347	-14.4252	0.22	1.00E-05
LINC01985	0.00021	0.000906	-13.3581	0.105	1.00E-05
RGSL1	7.39E-07	7.98E-06	-13.2877	0.1	1.00E-05
TERB2	2.39E-08	5.01E-07	-12.7731	0.07	1.00E-05
SPATA46	2.56E-05	0.000154	-12.2877	0.05	1.00E-05
RPS4XP5	3.15E-05	0.000183	-12.1357	0.045	1.00E-05
GLYATL1P4	0.017867	0.03897	-11.9658	0.04	1.00E-05
NAV2-AS4	4.08E-07	4.86E-06	-11.9658	0.04	1.00E-05
JAKMIP2-AS1	2.80E-05	0.000166	-11.5507	0.03	1.00E-05
DNMT3L	1.33E-07	1.96E-06	-5.6366	0.99501	0.02
HSD17B13	1.12E-06	1.11E-05	-4.3045	14.81978	0.75
SPP2	3.88E-19	6.74E-16	-4.0415	99.45941	6.03998
LINC00844	0.000131	0.000607	-3.8316	8.68489	0.61
CYP2A6	9.19E-07	9.50E-06	-3.6468	152.6268	12.18496
UROC1	3.31E-08	6.47E-07	-3.6329	7.50499	0.60499
CYP3A4	2.11E-08	4.49E-07	-3.5817	53.99556	4.50994
CYP2E1	7.45E-06	5.49E-05	-3.5733	1473.412	123.7812
SLCO1B7	4.70E-05	0.000256	-3.5236	0.115	0.01
FAM99A	7.11E-10	2.81E-08	-3.5235	2.87499	0.25001
SLC10A1	1.21E-10	7.31E-09	-3.4719	43.93909	3.9601
AQP9	1.56E-12	2.37E-10	-3.4311	108.6692	10.07505
TTC36	1.01E-06	1.02E-05	-3.3932	8.3524	0.795
GLYAT	9.15E-09	2.26E-07	-3.3499	57.96111	5.68501
LINC01018	1.09E-05	7.54E-05	-3.3176	6.7798	0.68001
HGFAC	0.000266	0.001111	-3.2597	30.83958	3.22
CRYAA	8.98E-07	9.36E-06	-3.1753	5.46506	0.60499
RHBG	7.62E-05	0.000386	-3.1712	5.85497	0.65
SNTG1	0.000288	0.001185	-3.1699	0.09	0.01
CRYAA2	5.54E-06	4.28E-05	-3.1567	8.65009	0.96997
CYP7A1	2.90E-09	9.06E-08	-3.1219	18.75996	2.15501
RTP3	9.87E-08	1.56E-06	-3.1197	21.59997	2.48501
AKR1C6P	9.92E-09	2.40E-07	-3.1057	1.97998	0.23001
ELFN1	7.42E-10	2.91E-08	-3.0372	5.49998	0.67
CHRNA4	0.004606	0.012444	-3.0256	1.14002	0.14
GCGR	4.36E-07	5.15E-06	-3.0237	12.89003	1.58501
PCDH20	0.000739	0.002632	-2.9542	0.155	0.02
HEPACAM	0.001041	0.003498	-2.8745	0.11	0.015
TDGF1	3.66E-08	7.04E-07	-2.8656	5.94011	0.81499
CYP1A2	0.000562	0.002086	-2.8509	0.505	0.07
ZNF648	7.07E-08	1.18E-06	-2.848	0.18	0.025
IGF2-AS	0.001356	0.004382	-2.8074	0.07	0.01
MLIP-AS1	0.000292	0.001199	-2.8074	0.07	0.01

UGT1A4	4.18E-06	3.37E-05	-2.7627	75.841	11.17515
THRSP	0.005851	0.015278	-2.7619	9.05491	1.33497
CYP8B1	1.55E-06	1.46E-05	-2.759	49.68596	7.33984
FAM245A	4.96E-07	5.76E-06	-2.7425	0.43501	0.065
LINC01485	3.03E-08	6.05E-07	-2.7238	96.08471	14.54506
LECT2	1.38E-10	7.86E-09	-2.7144	72.95069	11.11523
DHRS2	0.000867	0.003008	-2.6898	10.13028	1.57
CD300LG	2.30E-08	4.84E-07	-2.6781	0.16	0.025
ADH4	4.54E-09	1.30E-07	-2.6679	215.6273	33.92973
SLCO1B3	0.00034	0.001365	-2.6634	5.28983	0.83498
SLC22A1	1.75E-08	3.86E-07	-2.6497	55.255	8.80519
CYP2A13	2.53E-06	2.18E-05	-2.6439	0.25001	0.04
GFRA1	1.52E-05	1.00E-04	-2.6162	4.65992	0.76001
ADH1C	4.52E-06	3.60E-05	-2.6132	271.1166	44.30944
HORMAD2-AS1	2.49E-06	2.16E-05	-2.6127	13.64036	2.23004
ADH7	5.25E-05	0.000282	-2.585	0.06	0.01
DRD1	2.14E-09	7.06E-08	-2.585	0.06	0.01
CYP2A7	3.16E-05	0.000184	-2.5796	2.69	0.45001
LINC02754	2.73E-07	3.57E-06	-2.5663	8.44004	1.42499
SEC14L3	3.78E-06	3.10E-05	-2.5546	0.235	0.04
CCDC196	7.98E-05	0.000401	-2.5443	0.35	0.06
MOGAT2	2.80E-07	3.63E-06	-2.539	8.19493	1.41002
CNDP1	0.002424	0.007202	-2.4919	1.12502	0.2
OPRPN	0.000347	0.001388	-2.4894	0.365	0.065
LINC01780	8.43E-07	8.91E-06	-2.4854	0.7	0.125
RANBP3L	5.25E-09	1.47E-07	-2.4854	0.28	0.05
C3orf85	7.91E-05	0.000398	-2.4802	1.39498	0.25001
APOC4	6.71E-13	1.15E-10	-2.4592	410.7096	74.68592
CYP3A7-CYP3A51P	3.86E-07	4.64E-06	-2.4521	0.98501	0.18
TRPM8	1.26E-08	2.95E-07	-2.4519	8.18	1.49501
GPLD1	6.43E-11	4.41E-09	-2.441	6.4349	1.18501
CYP2C8	1.50E-07	2.17E-06	-2.4267	171.0021	31.80485
HRG	9.31E-09	2.29E-07	-2.3988	1056.79	200.3867
IGF2	0.000876	0.003033	-2.3917	35.37005	6.73997
MTND5P1	8.00E-08	1.30E-06	-2.3692	0.155	0.03
RB1-DT	0.000108	0.000519	-2.3656	0.33499	0.065
FOLH1B	7.28E-05	0.000371	-2.3374	0.93499	0.185
SLC1A2	3.73E-06	3.07E-05	-2.3349	3.355	0.66498
SRD5A2	9.38E-06	6.64E-05	-2.3274	1.30502	0.26001
LINC00402	1.27E-07	1.91E-06	-2.3219	0.05	0.01
GYS2	6.81E-09	1.79E-07	-2.3202	8.23999	1.64996
CFHR4	1.05E-07	1.63E-06	-2.316	17.08005	3.43003
DSG1	3.13E-06	2.63E-05	-2.2977	0.88499	0.18
CCL16	4.42E-09	1.28E-07	-2.2971	61.30919	12.47472

ASPDH	3.72E-11	2.83E-09	-2.2769	42.67085	8.80481
LINC01093	0.000474	0.00181	-2.2713	0.69999	0.145
LINC02037	2.31E-08	4.86E-07	-2.2701	0.82	0.17
FMO3	2.71E-14	9.42E-12	-2.2695	191.6721	39.7539
MTND4P20	1.06E-06	1.07E-05	-2.2517	7.21501	1.515
TTR	5.96E-10	2.44E-08	-2.2505	2464.078	517.8323
ACADL	7.48E-05	0.00038	-2.25	3.52009	0.73999
GLYATL1	7.32E-11	4.83E-09	-2.2483	58.27476	12.26516
RTL9	0.000632	0.00231	-2.2479	0.095	0.02
PGLYRP2	5.51E-10	2.29E-08	-2.2478	67.18328	14.14504
RDH16	1.15E-08	2.74E-07	-2.2429	47.62015	10.06003
IGSF23	1.18E-08	2.80E-07	-2.2375	10.96483	2.32505
FABP4	6.70E-09	1.77E-07	-2.2241	11.0498	2.36505
CD5L	0.000307	0.001255	-2.2146	1.23001	0.265
NOTUM	1.35E-05	9.06E-05	-2.214	5.15012	1.11
FAM99B	1.58E-06	1.47E-05	-2.2016	0.345	0.075
UGT1A2P	1.35E-05	9.05E-05	-2.195	0.435	0.095
FNDC5	3.61E-06	2.98E-05	-2.1782	5.68004	1.25502
DEFB132	0.001034	0.003483	-2.1699	0.135	0.03
LCN6	3.00E-05	0.000176	-2.1699	0.09	0.02
LINC01344	1.08E-10	6.69E-09	-2.1699	0.045	0.01
PRR18	1.92E-10	9.98E-09	-2.1654	1.57	0.34999
GNMT	1.94E-09	6.47E-08	-2.1653	43.55588	9.70989
UPB1	2.05E-15	1.35E-12	-2.1653	90.20942	20.11033
LINC02499	3.93E-11	2.97E-09	-2.1632	9.02516	2.015
SLC22A7	1.01E-07	1.59E-06	-2.1607	105.8603	23.67521
DNMT3L-AS1	8.01E-06	5.84E-05	-2.1565	0.535	0.12
AFM	6.44E-13	1.12E-10	-2.152	74.80378	16.83033
LGR5	0.017986	0.039179	-2.1468	0.46499	0.105
SLC25A47	1.07E-05	7.40E-05	-2.1167	19.04002	4.39003
CYP1A1	0.008463	0.020885	-2.1155	1.49497	0.34499
SLC2A2	8.99E-17	8.18E-14	-2.0722	146.6609	34.87541
CFHR5	5.55E-06	4.29E-05	-2.0712	30.29915	7.21011
CYP2C9	1.27E-09	4.59E-08	-2.0581	237.8538	57.11683
ABCB11	2.77E-06	2.37E-05	-2.0495	3.70505	0.89499
LINC02708	4.70E-05	0.000256	-2.0473	0.61999	0.15
CYP4F2	4.32E-09	1.25E-07	-2.0444	42.26048	10.24505
SLC27A5	1.89E-11	1.71E-09	-2.0416	291.5967	70.82622
TAT	7.45E-05	0.000379	-2.0346	110.7852	27.0395
ADH1B	2.60E-07	3.43E-06	-2.0312	416.7446	101.9575
SLC39A5	8.42E-06	6.08E-05	-2.0289	76.98921	18.86504
LINC01549	3.74E-06	3.08E-05	-2.0286	0.51	0.125
HPD	0.003139	0.008959	-2.0211	563.5421	138.8418
RIPPLY1	5.27E-05	0.000283	-2.0148	1.47502	0.365

HNF4A-AS1	8.43E-06	6.08E-05	-2.009	5.61495	1.39497
AKR1D1	1.74E-08	3.84E-07	-2.0089	14.65028	3.63992
CFAP57	3.06E-05	0.000179	-2	0.48	0.12
DSCAS	0.022965	0.048176	-2	0.06	0.015
PACRG-AS1	0.002307	0.006914	-2	0.02	0.005
PRAMENP	9.04E-05	0.000445	-2	0.04	0.01
SLIT3-AS2	1.87E-07	2.61E-06	-2	0.04	0.01
CFHR3	4.77E-06	3.76E-05	-1.9937	46.97493	11.79513
FETUB	1.51E-05	9.98E-05	-1.9707	39.23503	10.01007
ACSM2A	2.88E-10	1.38E-08	-1.9696	113.2124	28.90526
TPRG1-AS1	5.23E-07	6.03E-06	-1.9619	3.17496	0.815
TMSB4Y	0.008946	0.021882	-1.9615	0.37	0.095
HORMAD2	3.82E-05	0.000215	-1.9541	0.30999	0.08
REN	3.44E-07	4.25E-06	-1.9526	0.59998	0.155
IL27	6.12E-09	1.65E-07	-1.9477	4.33992	1.125
FGF21	0.021577	0.045703	-1.9438	25.31452	6.58
BCHE	5.25E-08	9.34E-07	-1.942	22.76532	5.92491
ACSM5	2.03E-06	1.83E-05	-1.9407	33.13035	8.63012
F9	5.59E-06	4.31E-05	-1.9288	41.50049	10.90009
LINC00570	2.47E-06	2.15E-05	-1.926	0.095	0.025
ADH1A	2.17E-10	1.10E-08	-1.9224	353.1539	93.17045
ANO1	4.70E-07	5.49E-06	-1.9159	13.49024	3.57497
DNMBP-AS1	2.51E-05	0.000152	-1.9085	1.14499	0.305
MBL2	2.46E-06	2.14E-05	-1.8874	15.09502	4.07996
SSTR1	0.001886	0.005812	-1.8779	2.09495	0.57001
UGT2B27P	2.32E-06	2.05E-05	-1.8768	1.06501	0.28999
NR1I3	1.19E-09	4.34E-08	-1.8747	71.01412	19.36489
DAO	3.94E-10	1.78E-08	-1.8473	28.84022	8.01492
CYP4A22	1.14E-11	1.16E-09	-1.8371	30.6551	8.58009
TCP10L	0.002974	0.008555	-1.8357	5.01498	1.40499
ESR1	6.93E-07	7.58E-06	-1.826	0.585	0.165
CYP3A7	0.000164	0.000733	-1.8176	25.41477	7.2102
CUX2	1.34E-07	1.97E-06	-1.8148	3.87008	1.10003
ADORA2BP1	0.004791	0.012869	-1.8129	0.64999	0.185
ERLNC1	0.000157	0.000708	-1.8074	0.07	0.02
GDPD4	6.79E-06	5.08E-05	-1.8074	0.21	0.06
LINC00885	0.002854	0.008271	-1.8074	0.07	0.02
NACAP8	0.005037	0.013459	-1.8074	0.07	0.02
TRAPPC13P1	0.003389	0.009573	-1.8074	0.07	0.02
PLIN1	2.05E-07	2.80E-06	-1.8038	3.05492	0.87499
HAO2	0.001031	0.003472	-1.8004	10.30992	2.95994
SLC6A1	2.80E-12	3.80E-10	-1.79	31.20932	9.02496
IGFALS	0.00021	0.000906	-1.7839	5.78494	1.67998
ABCA8	2.18E-05	0.000135	-1.7604	3.92994	1.15999

AZGP1P2	3.92E-06	3.20E-05	-1.7549	0.405	0.12
LINC02362	3.43E-07	4.25E-06	-1.7549	4.05008	1.20001
PDK4	3.43E-07	4.25E-06	-1.7475	29.93495	8.91525
PCP4L1	0.018681	0.040435	-1.7386	1.43501	0.43001
LRRC3-DT	4.41E-05	0.000243	-1.737	0.1	0.03
QRFPR	0.000233	0.000991	-1.737	0.05	0.015
CYP3A43	4.62E-07	5.41E-06	-1.7346	1.01498	0.305
ELOVL2-AS1	6.01E-07	6.75E-06	-1.7308	0.78	0.235
SERPINA4	1.19E-16	1.03E-13	-1.7307	140.0709	42.20442
PCK1	0.000321	0.001306	-1.7287	107.4647	32.42496
CES3	1.34E-10	7.69E-09	-1.7241	10.76985	3.25997
TMPRSS6	1.04E-12	1.68E-10	-1.7151	72.54408	22.09537
SERPINC1	1.39E-14	5.77E-12	-1.7144	1933.578	589.2064
RNU1-70P	2.02E-12	2.94E-10	-1.7138	95.37046	29.07451
CHCHD4P3	3.09E-07	3.94E-06	-1.7137	0.41	0.125
IYD	5.70E-10	2.36E-08	-1.7055	3.17996	0.97502
SEC14L2	2.76E-10	1.33E-08	-1.701	63.7728	19.61492
CYP4A11	2.00E-09	6.66E-08	-1.6968	138.6661	42.77417
ETNK2	9.15E-08	1.45E-06	-1.6954	58.73495	18.13547
RALGPS2-AS1	1.51E-11	1.45E-09	-1.6933	0.75999	0.235
AOC4P	3.75E-08	7.16E-07	-1.6917	5.95999	1.84499
EHHADH	3.02E-11	2.46E-09	-1.6882	58.81172	18.24978
ACSM2B	7.36E-10	2.90E-08	-1.6838	232.4446	72.34886
HSD17B6	9.08E-09	2.25E-07	-1.6833	307.5209	95.75101
CTD-3080P12.3	8.59E-05	0.000426	-1.6829	0.61	0.18999
TPPP2	7.67E-10	2.99E-08	-1.6812	0.92999	0.29
NAT2	0.008428	0.020823	-1.6794	3.07493	0.96
UGT2B10	1.45E-06	1.38E-05	-1.6792	80.99224	25.28994
PLGLA	2.89E-06	2.45E-05	-1.6682	5.08495	1.59999
PKLR	1.10E-10	6.76E-09	-1.6646	52.56125	16.57991
TDO2	0.011	0.025942	-1.6638	86.64262	27.34433
TENM2	0.000821	0.002867	-1.663	0.19	0.06
A1BG	3.95E-12	4.93E-10	-1.6625	1195.156	377.5333
THBS4	0.00138	0.004447	-1.6599	4.645	1.47
TTPA	6.33E-08	1.08E-06	-1.6574	18.07501	5.72987
FAM169A	9.30E-06	6.59E-05	-1.6548	0.73999	0.235
GREB1	2.22E-05	0.000137	-1.6547	2.33	0.74
CCND2P1	0.00035	0.001399	-1.6527	4.68487	1.49001
CCL25	0.000893	0.003077	-1.6521	0.99003	0.315
HLF	3.30E-09	9.98E-08	-1.6505	16.21497	5.16507
SLC13A5	2.01E-08	4.33E-07	-1.6481	72.87298	23.25021
HPR	9.93E-07	1.01E-05	-1.6447	241.3262	77.1814
CYP26A1	0.000114	0.000539	-1.6439	0.375	0.12
NUGGC	7.53E-07	8.08E-06	-1.6378	2.22497	0.715

CYP27A1	1.30E-14	5.77E-12	-1.634	264.1045	85.0951
ZNF385B	3.73E-06	3.07E-05	-1.6338	2.85494	0.91999
SLC22A25	7.07E-07	7.70E-06	-1.6329	2.44998	0.78997
ZG16	6.01E-06	4.58E-05	-1.6314	1.58003	0.51
HCG14	7.05E-06	5.25E-05	-1.628	0.17	0.055
ABCA9	1.69E-11	1.59E-09	-1.6215	0.80001	0.26
PFKFB1	8.47E-06	6.11E-05	-1.6155	6.68	2.18001
ABAT	1.55E-08	3.49E-07	-1.6154	59.4718	19.40999
LINC01702	0.001545	0.004904	-1.6122	5.35012	1.74999
LAMA5-AS1	0.010374	0.0247	-1.6097	5.28004	1.73004
LINC01595	4.49E-05	0.000246	-1.6052	1.08003	0.355
HAPLN4	1.98E-06	1.79E-05	-1.6041	0.38	0.125
SLC17A4	1.96E-10	1.01E-08	-1.6013	15.10974	4.97989
TRIM80P	1.19E-09	4.35E-08	-1.5999	0.485	0.16
PIPOX	1.05E-13	2.75E-11	-1.5939	146.931	48.67492
HS1BP3-IT1	1.37E-07	2.01E-06	-1.5919	3.13498	1.04
GLS2	0.018411	0.039934	-1.5876	8.50522	2.82999
G6PC1	3.25E-09	9.88E-08	-1.5871	134.1487	44.6509
FXYD1	7.36E-05	0.000375	-1.586	86.55398	28.83028
AOX2P	0.010549	0.025048	-1.585	0.03	0.01
CCDC187	0.005139	0.013679	-1.585	0.15	0.05
CHN2-AS1	0.000442	0.001706	-1.585	0.135	0.045
COL25A1	3.85E-05	0.000217	-1.585	0.03	0.01
HSD3B2	0.00165	0.005189	-1.585	0.06	0.02
JAKMIP2	3.22E-06	2.70E-05	-1.585	0.36001	0.12
LINC01744	0.016249	0.035962	-1.585	0.03	0.01
LINGO4	1.64E-06	1.52E-05	-1.585	0.24	0.08
LIPJ	3.36E-05	0.000193	-1.585	0.03	0.01
RPL7P60	0.013849	0.031465	-1.585	0.09	0.03
VWA8-AS1	2.08E-06	1.86E-05	-1.585	0.03	0.01
ZFHX4-AS1	0.001168	0.003863	-1.585	0.09	0.03
OTC	2.96E-08	5.94E-07	-1.5816	56.98601	19.04017
CYP2B6	4.57E-08	8.35E-07	-1.5706	16.46987	5.54499
NECAB2	0.007307	0.0185	-1.5704	5.91006	1.98997
ACOT12	2.07E-08	4.44E-07	-1.5638	16.96954	5.73995
BTNL9	3.60E-10	1.65E-08	-1.5634	2.33494	0.79001
IFNLR1	1.40E-10	7.95E-09	-1.5556	2.19	0.745
MYCL	2.75E-12	3.78E-10	-1.5527	6.86487	2.33999
ALDH1L1	0.000258	0.001081	-1.5495	113.4699	38.76434
GATM	1.22E-13	3.14E-11	-1.5454	389.3972	133.4069
GBP7	0.002643	0.007751	-1.545	6.58	2.25501
CHAD	1.38E-13	3.38E-11	-1.5392	11.30512	3.88994
NLRP6	0.000256	0.001073	-1.5377	0.45	0.155
FCN2	0.000456	0.001754	-1.5361	0.29	0.1

SLC2A12	0.000934	0.003197	-1.5318	1.19997	0.415
C9orf152	0.0032	0.009108	-1.5306	0.39001	0.135
AOX3P	0.00129	0.004194	-1.5236	0.115	0.04
APOA1	4.26E-08	7.88E-07	-1.5232	7312.626	2544.151
SIGLEC15	0.008951	0.02189	-1.522	0.56	0.195
DNASE1L3	8.16E-09	2.07E-07	-1.5143	10.06993	3.52503
CYP3A5	4.58E-10	1.99E-08	-1.5112	141.6405	49.69027
RORC	2.43E-14	8.95E-12	-1.5064	48.78081	17.16995
DIO1	0.002561	0.007547	-1.506	57.16042	20.12504
GSTA1	0.000262	0.001097	-1.5059	766.0888	269.753
BHMT	6.40E-05	0.000334	-1.504	63.4904	22.38473
HSD11B1	0.003327	0.009415	-1.5013	70.50115	24.90422
LINC01127	2.31E-09	7.51E-08	-1.5009	1.50001	0.53
ACSL6	0.000201	0.000872	-1.4963	1.73498	0.615
AMD1P4	0.010784	0.025505	-1.4854	0.07	0.025
RPL17P11	0.005742	0.015019	-1.4854	0.14	0.05
RPL7AP15	7.94E-05	0.000399	-1.4854	0.07	0.025
CYP2D6	5.97E-06	4.55E-05	-1.4834	194.2468	69.46977
PRODH2	6.43E-09	1.71E-07	-1.4823	65.60431	23.48041
PCK2	1.31E-11	1.28E-09	-1.482	181.076	64.82441
SLC13A2	0.003076	0.008806	-1.4806	0.60001	0.215
CTNNA3	1.41E-05	9.38E-05	-1.478	0.195	0.07
F12	4.38E-13	8.21E-11	-1.4772	238.1584	85.54035
FCAMR	0.006771	0.017354	-1.4754	0.57001	0.205
ENPP3	0.01301	0.029922	-1.4739	0.75	0.27
TREH	0.000547	0.002043	-1.4695	0.89999	0.32499
CPED1	0.000116	0.000548	-1.4655	2.95494	1.07003
APOM	2.69E-08	5.53E-07	-1.4649	354.3914	128.384
CLEC3B	3.31E-08	6.47E-07	-1.4638	15.57027	5.64494
INHBC	6.31E-07	7.00E-06	-1.4631	21.56003	7.81994
LINC02637	5.09E-10	2.16E-08	-1.4626	3.95497	1.43497
GABBR2	0.023149	0.048519	-1.4594	0.055	0.02
GNAT1	0.000531	0.001993	-1.4594	0.165	0.06
LINC00222	0.001702	0.005323	-1.4594	0.11	0.04
OMD	0.008655	0.021291	-1.4594	0.055	0.02
SPDYC	8.20E-09	2.08E-07	-1.4552	0.84998	0.31
ARID3C	1.06E-08	2.53E-07	-1.4484	0.65499	0.24
SLC6A13	7.11E-05	0.000364	-1.4388	2.85999	1.055
SHBG	0.000158	0.00071	-1.4373	14.05491	5.18996
HEPN1	0.001214	0.003993	-1.433	0.135	0.05
CCDC170	5.43E-06	4.21E-05	-1.4289	0.87498	0.32499
NADK2-AS1	0.000178	0.000786	-1.4288	0.175	0.065
SLC6A12	3.36E-07	4.18E-06	-1.4282	18.29979	6.79991
CDO1	2.85E-13	6.18E-11	-1.4278	106.9792	39.76383

SHD	7.49E-06	5.51E-05	-1.4251	7.46518	2.78001
SLC16A11	6.80E-06	5.08E-05	-1.4242	9.5	3.54005
CCL14	2.01E-08	4.33E-07	-1.4228	9.62525	3.59004
GPER1	5.82E-09	1.59E-07	-1.422	8.6149	3.215
ADRB2	3.08E-08	6.14E-07	-1.4203	1.365	0.51001
ADH6	5.53E-13	9.89E-11	-1.4194	73.34314	27.41972
CALML3-AS1	2.85E-06	2.42E-05	-1.415	0.08	0.03
BANF1P2	4.37E-10	1.91E-08	-1.4139	2.105	0.79001
KCNJ8	5.86E-08	1.01E-06	-1.4108	14.53006	5.46497
DCXR	2.45E-07	3.26E-06	-1.4103	660.3705	248.456
PHYHD1	0.012572	0.029067	-1.4102	17.27515	6.49996
APOF	7.40E-06	5.46E-05	-1.4094	16.92005	6.36997
CTXND1	1.89E-07	2.63E-06	-1.4027	3.11994	1.18
DCXR-DT	0.000205	0.000889	-1.402	4.76988	1.805
FTCD	2.15E-11	1.89E-09	-1.4015	224.4397	84.95954
BDH1	3.72E-13	7.48E-11	-1.3966	127.1009	48.2756
KNG1	1.62E-11	1.53E-09	-1.3916	1262.398	481.1416
SLC22A10	5.66E-06	4.36E-05	-1.3911	6.25511	2.38498
SLC38A4	1.96E-07	2.71E-06	-1.3895	61.89182	23.62449
LINC02259	6.93E-07	7.58E-06	-1.387	0.33999	0.13
KRTAP5-6	5.56E-08	9.71E-07	-1.3857	0.405	0.155
CSAD	7.59E-14	2.12E-11	-1.3848	22.34017	8.55483
SLC13A3	0.006922	0.017678	-1.3804	6.0398	2.32001
GPAM	2.96E-10	1.41E-08	-1.3781	35.96099	13.83521
TUSC8	2.34E-07	3.15E-06	-1.3769	2.80501	1.08003
C7	0.00502	0.013418	-1.3651	8.56491	3.32497
AHSG	2.45E-07	3.26E-06	-1.3607	1677.168	653.1004
PPP1R1A	0.000963	0.003276	-1.3557	59.1796	23.12448
LINC02675	3.13E-05	0.000182	-1.3508	1.13501	0.44501
CLDN14	5.06E-09	1.42E-07	-1.3494	13.78509	5.41003
LRRC3	1.32E-07	1.95E-06	-1.3492	4.00001	1.57003
LINC02029	0.002209	0.006654	-1.3479	0.27999	0.11
SERPINF2	2.24E-11	1.94E-09	-1.3448	525.0417	206.7072
EXOC3L4	3.24E-07	4.06E-06	-1.3326	24.27988	9.6402
AADAC	3.97E-10	1.78E-08	-1.3291	155.1835	61.76375
CYP4F3	9.36E-08	1.48E-06	-1.3289	43.04636	17.13502
LPA	2.89E-08	5.83E-07	-1.3264	1.60499	0.63999
SORD	3.50E-09	1.05E-07	-1.3258	95.58995	38.13424
HJV	1.16E-07	1.76E-06	-1.3241	122.6978	49.00448
CA4	0.000214	0.000922	-1.322	0.40001	0.16
ADRA1A	0.000555	0.002067	-1.3219	0.72498	0.29
CPS1-IT1	0.002927	0.00844	-1.3219	0.05	0.02
HMGB3P10	0.00011	0.000525	-1.3219	0.1	0.04
LARGE1-AS1	0.000347	0.001389	-1.3219	0.25	0.1

LYPLAL1-DT	0.003224	0.009165	-1.3219	0.075	0.03
ITIH1	3.45E-12	4.45E-10	-1.3179	877.9518	352.1623
HOMER2	8.15E-07	8.62E-06	-1.3159	23.41502	9.40495
SAA4	4.18E-06	3.37E-05	-1.3142	232.2195	93.38474
SLC27A2	8.50E-07	8.96E-06	-1.3132	54.54537	21.94992
SHLD2P3	3.72E-10	1.69E-08	-1.3093	0.57	0.23001
SERPINA11	0.000168	0.000751	-1.3087	63.30451	25.55518
FMO4	1.52E-08	3.42E-07	-1.3084	11.37985	4.59497
GMNC	0.005712	0.014955	-1.2996	0.48	0.195
NCMAP-DT	4.61E-05	0.000252	-1.2996	0.16	0.065
ATF7IP2	6.29E-06	4.75E-05	-1.2885	26.1747	10.71518
APOH	2.71E-14	9.42E-12	-1.2881	4204.626	1721.806
WNK3	1.94E-07	2.69E-06	-1.288	1.05003	0.43001
DBH	3.80E-08	7.23E-07	-1.2872	0.71998	0.295
ABCC9	0.000246	0.001036	-1.2867	2.52506	1.035
AR	1.66E-07	2.37E-06	-1.2848	5.94492	2.43998
ABCB4	1.55E-06	1.46E-05	-1.2842	52.98358	21.75538
CA14	0.000499	0.001893	-1.2831	2.385	0.98002
EVPLL	0.013197	0.030259	-1.2801	0.255	0.105
LINC00933	1.72E-05	0.00011	-1.2801	0.17	0.07
EPHX2	3.12E-12	4.16E-10	-1.2788	56.72957	23.38043
SLCO1B1	6.04E-07	6.76E-06	-1.2782	43.81502	18.06512
DPYS	2.54E-07	3.37E-06	-1.2768	168.7864	69.66126
LINC02028	2.24E-07	3.03E-06	-1.2761	1.55	0.64
PANK1	7.28E-14	2.08E-11	-1.2759	17.12008	7.07018
EPHA1	0.000571	0.002111	-1.2757	16.64502	6.87491
DBH-AS1	4.15E-10	1.85E-08	-1.2752	5.98996	2.47494
UGT2B15	0.001104	0.003682	-1.2745	136.3299	56.35562
MGC32805	6.82E-05	0.000353	-1.2727	1.65501	0.685
ZBTB16	0.001315	0.004264	-1.2671	3.16496	1.315
FOLH1	1.72E-05	0.000111	-1.2666	6.45994	2.68501
CFHR2	2.74E-08	5.59E-07	-1.2634	264.4474	110.1559
ABCA6	3.53E-09	1.05E-07	-1.2602	20.18025	8.42501
STPG3	2.15E-06	1.92E-05	-1.2591	0.73001	0.305
CNGA1	6.95E-05	0.000358	-1.2586	3.90009	1.63003
TKFC	6.81E-09	1.79E-07	-1.2581	106.685	44.60474
FAXDC2	4.12E-10	1.83E-08	-1.2562	30.47969	12.76021
NR1I2	1.20E-05	8.20E-05	-1.255	10.58513	4.43499
LINC02565	0.001441	0.004621	-1.2538	0.155	0.065
SLC38A3	1.47E-13	3.54E-11	-1.2524	219.4649	92.12034
PALMD	1.50E-14	6.08E-12	-1.2496	18.63024	7.835
IGFBP2	0.001104	0.003682	-1.246	319.7378	134.8105
CRYL1	3.76E-13	7.48E-11	-1.2453	108.8159	45.90091
INSIG1	1.05E-05	7.33E-05	-1.2453	126.7214	53.45418

LINC02608	0.000981	0.003325	-1.2427	3.45504	1.45999
RHEBP2	3.72E-08	7.13E-07	-1.2419	12.49973	5.28501
ACADSB	3.28E-08	6.44E-07	-1.2415	39.77053	16.81969
CAT	1.98E-16	1.58E-13	-1.2408	175.3077	74.18158
C6	0.00012	0.000561	-1.2401	57.4497	24.32061
CLDN15	2.40E-05	0.000146	-1.2385	31.36947	13.29511
TMEM82	0.006183	0.016021	-1.2377	5.53002	2.34499
GLIS2-AS1	0.000377	0.001489	-1.237	1.15498	0.49
SERPINA5	3.94E-09	1.16E-07	-1.2362	243.0239	103.1597
ADORA2A-AS1	3.72E-13	7.48E-11	-1.2345	11.23505	4.77489
RGN	5.56E-10	2.31E-08	-1.2322	123.025	52.36836
CPS1	0.014177	0.03209	-1.2321	236.523	100.6896
LINC00671	8.46E-07	8.93E-06	-1.2294	0.33999	0.145
CYP2D7	0.00321	0.009132	-1.2279	22.10028	9.43515
LRCOL1	0.000495	0.001881	-1.2247	1.04	0.445
LINC01146	0.000281	0.001163	-1.2245	12.24496	5.24001
SLC17A2	1.78E-06	1.63E-05	-1.2237	14.61994	6.25988
SLC51A	0.001137	0.003775	-1.2236	63.01516	26.98447
DDX25	0.007448	0.018816	-1.2224	0.07	0.03
LINC01354	0.009673	0.023321	-1.2224	0.07	0.03
LINC01537	1.22E-06	1.20E-05	-1.2224	0.07	0.03
OGFR-AS1	0.009303	0.022575	-1.2224	0.14	0.06
PACRG	2.05E-06	1.85E-05	-1.2224	1.15502	0.495
CADM1	5.77E-08	9.98E-07	-1.2206	45.38447	19.47488
ALDH2	1.25E-10	7.40E-09	-1.2177	498.1717	214.1904
HS3ST3B1	2.24E-08	4.76E-07	-1.2177	6.20996	2.67004
C3P1	0.001497	0.004774	-1.2169	11.18006	4.80988
DMGDH	1.49E-08	3.38E-07	-1.2159	26.86481	11.56504
CGNL1	5.60E-11	4.01E-09	-1.2106	13.46977	5.82002
SORD2P	7.16E-09	1.85E-07	-1.206	44.93965	19.48012
STPG3-AS1	1.53E-06	1.44E-05	-1.2038	0.64499	0.28
F10-AS1	1.66E-05	0.000108	-1.1979	0.195	0.085
DHRS1	4.36E-12	5.24E-10	-1.1976	55.14942	24.0446
EXPH5	0.00129	0.004194	-1.1926	0.31999	0.14
APBA1	1.33E-05	8.95E-05	-1.1896	2.39496	1.05002
APOC3	3.81E-08	7.24E-07	-1.1877	8552.334	3754.574
EPHX1	5.79E-07	6.56E-06	-1.1848	1554.472	683.8007
GPIHBP1	1.07E-07	1.66E-06	-1.1848	1.57996	0.69501
TMEM220-AS1	2.37E-12	3.31E-10	-1.1848	5.36501	2.35999
ACSM5P1	0.00218	0.006581	-1.1844	0.125	0.055
TRMT112P4	0.002403	0.007149	-1.1844	0.75	0.33
ACE2	2.67E-02	5.48E-02	-1.1829	0.42	0.185
MYH7B	7.67E-07	8.19E-06	-1.1829	0.42	0.185
APOA5	2.25E-05	0.000139	-1.1827	100.1689	44.12563

SLC16A2	1.40E-08	3.21E-07	-1.1814	14.04999	6.19506
LEAP2	6.29E-06	4.75E-05	-1.175	108.4434	48.02636
HAO1	7.44E-08	1.23E-06	-1.1733	98.94958	43.87582
TMPRSS9	0.003311	0.009379	-1.171	1.60999	0.715
PROZ	5.00E-05	0.00027	-1.1707	9.90502	4.3999
ABHD12B	0.000118	0.000555	-1.1699	0.18	0.08
FBN3	0.005761	0.01506	-1.1699	0.135	0.06
GALR3	0.008181	0.02033	-1.1699	0.09	0.04
TMEM272	0.001083	0.003619	-1.1699	0.045	0.02
AZGP1P1	6.68E-08	1.13E-06	-1.1687	7.25009	3.22497
MTARC1	5.66E-06	4.36E-05	-1.1666	34.56993	15.40015
FABP1	0.000381	0.001502	-1.1656	2067.023	921.4284
SULT2A1	1.38E-05	9.18E-05	-1.1651	203.1209	90.58082
XDH	5.15E-05	0.000278	-1.1643	7.38516	3.29504
MPPED1	2.09E-05	0.00013	-1.1633	1.63497	0.72999
NCMAP	0.000648	0.002359	-1.1629	1.03002	0.46001
CIDEB	9.19E-07	9.50E-06	-1.1619	64.10334	28.65005
GOLGA8M	0.000102	0.000494	-1.1616	0.42501	0.18999
CASQ2	8.54E-06	6.14E-05	-1.1605	0.38	0.17
HMGCS2	7.38E-07	7.97E-06	-1.1598	565.4103	253.0663
F7	1.70E-10	9.27E-09	-1.1597	69.7609	31.22556
CES2	2.96E-09	9.21E-08	-1.1594	166.1153	74.37069
FTOP1	0.00012	0.00056	-1.1585	0.62499	0.27999
SCN4A	4.58E-07	5.37E-06	-1.1565	0.26749	0.12
SHLD2P1	4.07E-13	8.02E-11	-1.155	3.48503	1.56498
IAPP	6.46E-05	0.000337	-1.152	0.1	0.045
SEZ6	0.005603	0.01473	-1.152	0.1	0.045
TSPEAR-AS1	0.015155	0.033885	-1.152	0.69998	0.31499
SCNN1D	8.09E-08	1.31E-06	-1.1516	2.25503	1.01503
CD14	5.55E-08	9.70E-07	-1.1504	187.428	84.43874
HPX	3.42E-08	6.64E-07	-1.1486	1185.562	534.7481
GREB1L-DT	0.000868	0.00301	-1.1468	0.155	0.07
LGSN	0.018094	0.039361	-1.1468	0.155	0.07
RCL1	1.15E-09	4.24E-08	-1.1452	32.8898	14.87026
MIR99AHG	1.58E-05	0.000103	-1.145	4.655	2.10497
EGILA	3.06E-06	2.58E-05	-1.143	0.265	0.12
MLXIPL	4.06E-14	1.27E-11	-1.1417	114.2886	51.79948
F11	2.36E-07	3.17E-06	-1.1413	44.13937	20.01027
DGAT2	9.95E-08	1.56E-06	-1.141	79.16909	35.89992
AASS	0.000393	0.001544	-1.1378	4.22499	1.92
INHBA-AS1	9.87E-05	0.000479	-1.1375	0.11	0.05
ALAD	6.48E-14	1.88E-11	-1.1374	67.74443	30.79477
CPN2	1.42E-08	3.25E-07	-1.1371	79.79577	36.27993
CES4A	0.007966	0.019887	-1.1334	2.09499	0.95499

SLC16A12	0.00551	0.01453	-1.1312	0.23	0.105
NDUFA6-DT	3.78E-07	4.55E-06	-1.1273	6.21489	2.84497
MAMDC2-AS1	1.20E-05	8.18E-05	-1.1255	0.12	0.055
ACR	4.05E-06	3.28E-05	-1.1234	0.305	0.14
MAGOH2P	1.39E-05	9.28E-05	-1.1234	0.61	0.27999
AOX1	0.00022	0.000946	-1.1233	144.8756	66.50405
ECM2	4.46E-08	8.19E-07	-1.1223	7.63004	3.50497
PBLD	1.07E-07	1.65E-06	-1.1212	41.36483	19.0153
C4BPA	4.28E-05	0.000237	-1.1211	450.5249	207.1303
GPT2	0.007307	0.0185	-1.1167	40.00949	18.4502
GPR182	2.62E-05	0.000157	-1.1155	0.13	0.06
VIPR1	0.00042	0.001635	-1.1137	0.66	0.305
ADCY10	3.00E-05	0.000176	-1.1105	2.85003	1.31999
ETNPPL	0.00787	0.01968	-1.1105	17.01482	7.88017
RALGPS2	4.78E-11	3.52E-09	-1.1087	11.16006	5.17491
RHEBP1	1.36E-06	1.31E-05	-1.1077	11.40016	5.29007
TMEM88	2.81E-09	8.84E-08	-1.1021	4.75491	2.21499
RBP5	7.49E-05	0.00038	-1.102	104.705	48.77894
LIPC	5.31E-07	6.11E-06	-1.1019	63.8952	29.76923
LCAT	0.000512	0.001933	-1.1011	35.2453	16.4302
F13B	1.42E-07	2.07E-06	-1.1006	59.1549	27.58571
GPR17	4.13E-05	0.00023	-1.0995	0.15	0.07
ANG	9.00E-07	9.37E-06	-1.0976	444.6512	207.7856
CYB5A	6.07E-10	2.48E-08	-1.0963	758.1271	354.5783
ALDH6A1	3.15E-06	2.65E-05	-1.0939	33.32919	15.61478
GNA14	1.27E-07	1.89E-06	-1.0925	0.725	0.33999
ITPR2	3.50E-08	6.77E-07	-1.0905	10.61526	4.98501
TMC3-AS1	1.48E-09	5.20E-08	-1.0899	1.49	0.70001
NALT1	2.33E-05	0.000142	-1.089	0.58499	0.275
C6orf201	0.000189	0.000829	-1.0875	0.085	0.04
MIR325HG	0.008303	0.02056	-1.0875	0.085	0.04
SIAH2-AS1	0.000237	0.001008	-1.0875	0.17	0.08
PON3	2.29E-09	7.47E-08	-1.086	115.1091	54.2246
CBLN1	0.000488	0.001859	-1.0834	0.89	0.42
HOGA1	4.68E-05	0.000256	-1.0767	9.37508	4.44498
FAM166A	6.87E-05	0.000355	-1.074	0.2	0.095
TMEM220	7.59E-12	8.29E-10	-1.0719	23.55497	11.20509
PEMT	2.81E-09	8.84E-08	-1.0715	59.10909	28.1254
F11-AS1	3.84E-08	7.28E-07	-1.0712	2.91004	1.38498
DYNLT4	0.01305	0.029999	-1.0704	0.105	0.05
GOLGA6A	0.000848	0.00295	-1.0704	0.105	0.05
LINC02292	1.96E-05	0.000124	-1.0704	0.105	0.05
RAMP3	5.69E-05	0.000302	-1.0698	8.35505	3.9801
MLIP	0.000118	0.000556	-1.0696	8.18508	3.89989

HMGCS1	2.66E-05	0.000159	-1.0672	58.97353	28.14426
HCN3	3.24E-10	1.51E-08	-1.0665	10.1051	4.82491
STEAP4	0.001475	0.004718	-1.0641	0.80501	0.385
ITIH2	2.22E-09	7.30E-08	-1.063	736.4025	352.4677
FYB2	1.63E-06	1.51E-05	-1.0608	3.75505	1.8
MSMO1	8.76E-10	3.35E-08	-1.0559	124.9877	60.12077
MICU3	3.35E-06	2.79E-05	-1.0549	1.20503	0.58
PRAP1	6.29E-05	0.000329	-1.0547	756.1219	364.0018
ACSS3	0.003572	0.010018	-1.0543	15.17013	7.30495
UGT2B7	0.000613	0.002249	-1.0542	91.2763	43.95464
ALDH5A1	3.24E-15	1.93E-12	-1.0536	23.33046	11.24012
MPDZ	5.55E-08	9.70E-07	-1.0496	11.55014	5.57994
DAB1	0.01752	0.038327	-1.0495	0.445	0.215
SPATA21	0.011575	0.027121	-1.0489	0.29999	0.145
SNHG22	2.07E-06	1.86E-05	-1.0486	3.32002	1.60504
EMCN	2.49E-08	5.17E-07	-1.0457	4.48998	2.17503
LIME1	3.31E-05	0.000191	-1.0448	52.15521	25.27989
CCDC158	0.002065	0.00629	-1.0444	0.165	0.08
PEBP1P2	9.92E-06	6.96E-05	-1.0444	0.495	0.24
NAGS	6.79E-05	0.000351	-1.0418	12.26002	5.95499
KLF9	6.28E-08	1.07E-06	-1.0405	18.40968	8.94989
RNF165	0.004552	0.01232	-1.0395	0.185	0.09
HSD17B8	4.03E-12	4.94E-10	-1.0393	43.4555	21.14458
PPP1R1C	0.002231	0.00671	-1.0317	2.98498	1.46004
SORBS2	7.54E-07	8.08E-06	-1.0307	29.68439	14.53005
DBNDD1	0.001188	0.003918	-1.0283	13.13531	6.44013
HDC	0.001641	0.005166	-1.0275	0.265	0.13
PPP1R3G	0.006585	0.016929	-1.0259	2.81003	1.37998
NUTM2A	8.62E-06	6.20E-05	-1.0247	0.58999	0.28999
FBP1	6.07E-05	0.000319	-1.0238	122.4326	60.21507
HMGN2P47	0.000954	0.003251	-1.0217	0.33499	0.165
TFR2	4.06E-07	4.84E-06	-1.0203	241.9327	119.2783
PTGES3P2	0.004086	0.011248	-1.0192	1.135	0.56
BCO2	0.01307	0.030014	-1.0188	2.32004	1.14501
FMO5	6.07E-09	1.65E-07	-1.017	92.46493	45.69025
DHTKD1	2.72E-08	5.57E-07	-1.016	27.16026	13.43004
CPB2	1.40E-09	4.97E-08	-1.0158	292.6854	144.7467
GADD45G	1.87E-05	0.000118	-1.0155	50.47028	24.96546
ALDOB	0.000464	0.00178	-1.014	1238.222	613.1306
FBLN5	0.002221	0.006684	-1.0135	8.05511	3.99001
ALAS1	5.68E-09	1.56E-07	-1.0128	129.9858	64.41969
PPP1R3B	1.12E-07	1.73E-06	-1.0122	13.64492	6.76506
MTHFD1	1.60E-06	1.49E-05	-1.0118	89.36539	44.31972
LRRC2	7.84E-08	1.28E-06	-1.0113	1.925	0.955

RDH5	0.000161	0.000723	-1.0097	20.20484	10.03497
PAH	6.09E-08	1.05E-06	-1.0088	357.7558	177.7851
SLC25A25	9.45E-07	9.69E-06	-1.007	27.15054	13.50989
STARD5	4.82E-08	8.75E-07	-1.0067	7.57509	3.76988
KLKB1	4.04E-09	1.19E-07	-1.0042	29.45505	14.68494
NADK2	4.74E-11	3.50E-09	-1.0037	65.10554	32.47046
RPL23AP1	0.000754	0.002679	-1.0034	2.09501	1.04502
TLCD4	3.45E-10	1.59E-08	-1.0024	14.76497	7.37
TMEM150C	2.49E-08	5.17E-07	-1.0014	5.11483	2.55497
PECR	4.60E-08	8.41E-07	-1.001	62.73162	31.34441
ASTN1	0.001312	0.004255	-1	0.04	0.02
C1QTNF9	0.000182	0.000801	-1	0.06	0.03
CCDC73	1.51E-05	9.96E-05	-1	0.1	0.05
CYP2G1P	0.006474	0.016695	-1	0.02	0.01
CYP4Z1	0.003689	0.010293	-1	0.06	0.03
DCT	0.007478	0.018881	-1	0.02	0.01
FAM221B	0.000111	0.00053	-1	0.02	0.01
FMR1-AS1	0.002436	0.007227	-1	0.02	0.01
LINC00612	0.000493	0.001874	-1	0.06	0.03
LINC01482	0.001428	0.004582	-1	0.02	0.01
LINCR-0001	0.022265	0.046949	-1	0.12	0.06
LRRC3B	0.00212	0.006427	-1	0.06	0.03
LRRC77P	8.89E-05	0.000439	-1	0.12	0.06
MAPT-IT1	0.000929	0.003185	-1	0.02	0.01
NLRP14	0.020953	0.044552	-1	0.02	0.01
OR2C1	0.001681	0.005271	-1	0.02	0.01
OR7E29P	0.000156	0.000703	-1	0.06	0.03
PDE9A-AS1	0.000642	0.002339	-1	0.04	0.02
RNF217-AS1	0.004054	0.011181	-1	0.02	0.01
SAXO1	0.016136	0.035759	-1	0.02	0.01
SEPHS1P6	0.001259	0.004116	-1	0.06	0.03
SLC22A24	0.002307	0.006914	-1	0.02	0.01
TCTE1	1.84E-05	0.000117	-1	0.04	0.02
TDRD15	0.002085	0.006341	-1	0.02	0.01
USH2A	0.00062	0.00227	-1	0.17	0.085
